# Scoping review of public attitudes to government active travel interventions in the UK

**DOI:** 10.1136/bmjopen-2025-115598

**Published:** 2026-08-14

**Authors:** Nick Morris, Russell Jago, Helen P Russell, Ruth Salway, Kelly Morgan, Ruth Kipping

**Affiliations:** 1 Centre for Public Health, Bristol Medical School, University of Bristol, Bristol, UK; 2 Centre for the Development and Evaluation of Complex Interventions for Public Health Improvement (DECIPHer), School of Social and Spatial Sciences, Cardiff University, Cardiff, UK; 3 Population Health Sciences, Bristol Medical School, University of Bristol, Bristol, UK

**Keywords:** PUBLIC HEALTH, Attitude, Behavior

## Abstract

**Abstract:**

**Objective:**

Low physical activity levels are a public health concern. Active travel is a potential strategy to increase routine walking, wheeling and cycling across populations. However, governments in the UK have not yet achieved their active travel targets and gaining public acceptance for interventions can be challenging. To inform future policymaking, this review aims to synthesise evidence on public attitudes to active travel interventions in the UK.

**Design:**

A systematic scoping review.

**Data sources:**

Six sources (MEDLINE, Embase, CINAHL, Scopus, Web of Science and the Transport Research International Documentation) supplemented with a focused Google Scholar search.

**Eligibility criteria:**

Peer-reviewed evidence in English from 1999 to 2025 using any methods to analyse public attitudes to active travel interventions in any part of the UK.

**Data extraction and synthesis:**

After screening titles and abstracts, and then full-text review, we extracted data relevant to the research question. We produced descriptive statistics about the publications and narrative synthesis of public attitude themes, particularly how attitudes are formed and vary, and their role in policymaking.

**Results:**

We included 26 publications after screening 13 778 records. Publications were mostly published after 2021 (n=14). Physical changes to street design were the most common intervention type (n=16). Populations of interest included residents, commuters, schoolchildren and others exposed to local interventions. There was little focus on rural areas and generally limited longer-term, sustained data collection. Results suggest active travel was positively viewed overall but intervention delivery was challenging. How the public perceives intervention effectiveness and fairness were important but perceptions varied by public beliefs, characteristics and travel habits. Several policymaking and practice challenges were identified, including the complexities of gauging public consensus about interventions, underlying public cynicism and a widespread desire among drivers to maintain driving habits.

**Conclusions:**

Active travel interventions can be difficult to plan and implement due to public attitudes. Public engagement is an important part of any active travel interventions. There was a lack of evidence identified about the specific, practical strategies that policymakers can use to address engagement challenges. Further research is required to explore these issues to help tackle low levels of physical activity through active travel. However, this review provides some insight for policymakers about key public attitude considerations at any stage of designing and delivering active travel interventions.

**STRENGTHS AND LIMITATIONS OF THIS STUDY**

- Published protocol and a systematic search across six databases, and one other source, following the Preferred Reporting Items for Systematic Reviews and Meta-Analyses (PRISMA) extension for scoping reviews guidelines to ensure transparency and rigour.
- Two reviewers independently screened 30% of titles and abstracts, and 30% of full-text records.
- Covers the broad range of published evidence about active travel, a diverse topic spanning multiple public policy areas, travel modes, geographies and environmental and behavioural intervention types.
- Focus on the UK for policymaking reasons means that results cannot necessarily be transferred to other countries.
- The exclusion of grey literature, books, theses and conference proceedings due to resource constraints might have excluded some studies from the review.

## Introduction

### Background

 Populations globally are at risk of health problems, including coronary heart disease, diabetes, types of cancer and shorter life expectancy, due to low levels of physical activity.^1 2^ Nearly one-third of adults (31%) across the world did not meet recommended physical activity levels in 2020.^3^ Among high-income western countries, an average of 78.2% of adolescents were not active enough in 2016.^4^ In response to these challenges, many governments have implemented individual interventions and broader strategies to encourage active travel,^5^ which is routine physical activity through walking, wheeling and cycling for everyday purposes.^6^ The WHO recommends active travel as an achievable and relatively low-cost way to increase physical activity.^7^ Active travel is associated with cobenefits beyond increasing physical activity, including increased economic activity and cost savings, reductions in air pollution and carbon emissions, and physical and mental health and social well-being gains.^8^

Governments at national, regional and local levels can intervene to promote active travel^5 9^ with single or multicomponent interventions changing the natural or built environment^10^; and social and behavioural interventions, such as incentives to travel actively and restrictions to non-active travel.^9^ As well as being effective,^11^ public health interventions must also be deliverable, which includes being acceptable to the public.^12^ Acceptability refers to the degree to which the public and local communities think or feel interventions are appropriate.^13^ This can vary due to the nature of the intervention and which systems, behaviours or people the intervention affects.^14^ Variation in acceptability is also dynamic and involves complex interactions over time between the interventions, contexts and people involved.^15^ Active travel interventions can attract polarised public responses.^16^ Policymakers are also able to influence the acceptance of public health interventions^17^ which can help deliver and sustain interventions over time.^14^ However, without sufficient support from the public and local stakeholders, policymakers have been forced to modify, cancel or curtail active travel interventions at both planning and delivery stages.^18–20^

In recent decades the United Kingdom of England, Scotland, Wales and Northern Ireland has followed a process of devolution.^21^ This political process has involved individual nations and regions within the UK holding referenda on whether to transfer executive and legislative powers from central government in London. As a result, a growing number of geographic places have gained the ability to make independent decisions over a range of policy areas, including on active travel, public health and many other matters affecting populations’ everyday lives. The rationale for devolution is to make policy decisions more suitable and directly accountable to local people and places.^22^ This process of decentralising policymaking powers through devolution has led to diverging policy approaches^23^ and created a ‘giant policy laboratory’ within the UK.^24^ For example, legislative powers devolved to Wales in 2018^25^ directly enabled the Welsh Government to introduce a new default speed limit of 20 miles per hour in residential and built-up areas in 2023 as part of the Wales active travel strategy.^26^ In addition to policy variation, the devolution process also means that levels of acceptability for policies can vary between UK nations and regions.^27^

National governments within the UK, in England, Wales, Scotland and Northern Ireland, have undertaken or funded numerous active travel interventions over the past decade and many have strategies to increase active travel. However, audit bodies report that all four governments are falling short of their objectives to increase active travel overall^28–31^ though there have been increases in active travel participation and activity levels in some geographic areas, social groups and modes.^32 33^

Given these challenges and opportunities for active travel in the UK, there is a need for more evidence that policymakers can use to help gain public acceptance for changes to promote active travel. Existing reviews into the acceptability of public health interventions have combined active travel with other public health policy areas^34 35^ or focused on specific types of active travel intervention.^10^ As a result, attitudes towards the full range of active travel interventions might be insufficiently captured and analysed in depth. Public opposition to active travel interventions has been identified as a gap in the literature.^9^ Active travel policy is diverse, with relevance to public health, transport and town planning, and interventions include combinations of environmental and behavioural measures. Therefore, a dedicated focus on active travel is needed to explore the breadth and depth within active travel interventions and the responses to them.

### Research question and objectives

The research question for this scoping review was: what is the evidence on public attitudes towards government active travel interventions across the devolved nations and regions of the UK? This question requires systematic assessment of the scope of evidence in the UK,^36–39^ so a scoping review method was particularly appropriate for this topic given the need for an overview of potentially diverse evidence^37 38 40^ that covers multiple public policy areas, a range of contexts and interventions, and varied methods and designs used to quantify or explore the processes and meanings of attitudes.

The secondary aims were to:

- Summarise the evidence and data on public attitudes towards active travel interventions in the UK, and its nations and regions, since the advent of devolution in 1999.
- Identify themes in public support and opposition towards active travel interventions and any evidence about how these are formed and influenced.
- Explore similarities and differences between nations and regions; socioeconomic characteristics; and types of interventions and active travel modes.
- Examine how public attitudes can be a barrier or enabler to policymakers planning and delivering these interventions.

## Methods

### Registration

This study was registered on OSF on 24 February 2025 (https://doi.org/10.17605/OSF.IO/58A9X).

### Scoping review methodology

This study used the Preferred Reporting Items for Systematic Reviews and Meta-Analyses (PRISMA) guidance extension for Scoping Reviews (PRISMA-ScR)^39^ and drew on the Joanna Briggs Institute (JBI) scoping review manuals,^41^ and Arksey and O’Malley’s five-stage process for scoping reviews^42^:

### Research question and objectives (stage 1)

A draft question and objectives, based on identified evidence gaps, were shared during protocol development with three previous collaborators of the authors who had experience of academic, public health and local government roles. Our consultation purpose^43^ was to improve the potential utility of the review findings for people working in these roles. The feedback validated and refined the design, including improvements to the search strategy syntax covering the interventions concept.

### Identifying relevant studies (stage 2)

Criteria for identifying relevant publications, and for limiting the search, are summarised in table 1 and set out fully in the study protocol. The systematic search strategy drew on published literature,^9 44^ relevant Medical Subject Headings (MeSH terms) and advice from a subject librarian. A Medline search strategy (see online supplemental file 1) was piloted, refined and translated to the search interfaces used by Embase, CINAHL, Scopus, Web of Science and the Transport Research International Documentation (TRID). A focused Google Scholar search, following the focused search strategy described in Haddaway *et al*,^45^ was used for ‘difficult-to-locate literature’^46^ falling between disciplines.

**Table 1:** Summary criteria for identifying relevant publications

Area	Inclusion	Exclusion
Population and Context	Public and local communities in the UK as sole population of interest or in comparative studies with people from outside the UK.	Public solely outside the UK.
Concept 1: active travel	Modes requiring physical effort, such as walking, cycling or wheeling^6^ for everyday purposes, for entire journeys or journey stages.	Fully motorised modes, except as a mobility aid; and walking, cycling and wheeling solely for exercise or recreation.
Concept 2a: public attitudes	Attitudes of included population exposed to or affected by active travel interventions (including any related concepts such as perceptions, opinions, views and prior beliefs) that are objective or affective.^93^	Studies using attitudes solely/mainly to predict or explain active travel behaviour.
Concept 2b: active travel interventions	Interventions at any stage, including planned or hypothetical, intended by governments to increase active travel.^9 94 95^	Interventions with no specific aim to promote active travel as physical activity (e.g. policy only to reduce vehicular emissions).
Concept 3: UK devolution	Studies in UK since 1999, when the political devolution process started.^21^	Studies before 1999 or with most or all data pre-1999.
Source characteristics	English language; peer reviewed and using any combination of study designs, methods and data, including reviews.	Non-English language; grey literature, student theses, conference proceedings, unpublished studies; protocols, editorials and opinion pieces; and books.

### Study selection (stage 3)

Study selection started after the final search (5 March 2025). We removed duplicate results using EndNote (V.21.5) and the Covidence platform,^47^ which was used for screening. Two reviewers (NM and HPR) independently screened 30% of randomly selected titles and abstracts and resolved any conflicts, seeking a sufficiently high proportion of agreement^48^ in inclusion and exclusion decisions, before NM screened the remaining 70%. After this stage, the process was repeated with the remaining publication records’ full texts, with 30% independently screened by NM and HPR and the remaining 70% by NM. The proportion of independently screened records is consistent with other scoping reviews published in public health^49 50^ and in excess of the 20% threshold recommended for rapid reviews.^51^

### Data extraction or charting (stage 4)

NM developed an extraction form for data charting, which was based on the JBI evidence synthesis manual for scoping reviews^52^ with additional items specific to the research objectives, such as public attitude mechanisms and their relevance to policymakers. Two reviewers (NM and HPR) independently piloted the extraction form on three selected publications (~12% of included publications) that used qualitative, quantitative or mixed approaches. The pilot gauged whether the form charted sufficient depth and breadth to address the research question and objectives and NM then made amendments to improve the form and used it to independently chart the selected publications. Charted data were organised by research objective to enable synthesis and thematic analysis^53^ on the extracted findings. We tabulated categorical data, mainly about the characteristics of studies, interventions and populations of interest in each study. The thematic analysis of included study findings involved coding individual findings in each paper that related to the research question and objectives; combining these findings into initial themes; checking these themes against charted data; and further defining the themes.

### Collation, summarising and reporting (stage 5)

Using the charted data, we report descriptive statistics about the characteristics and a descriptive narrative summary of the findings in the included studies.

### Patient and public involvement

There was no patient or public involvement in this study.

## Results

**Figure 1:**
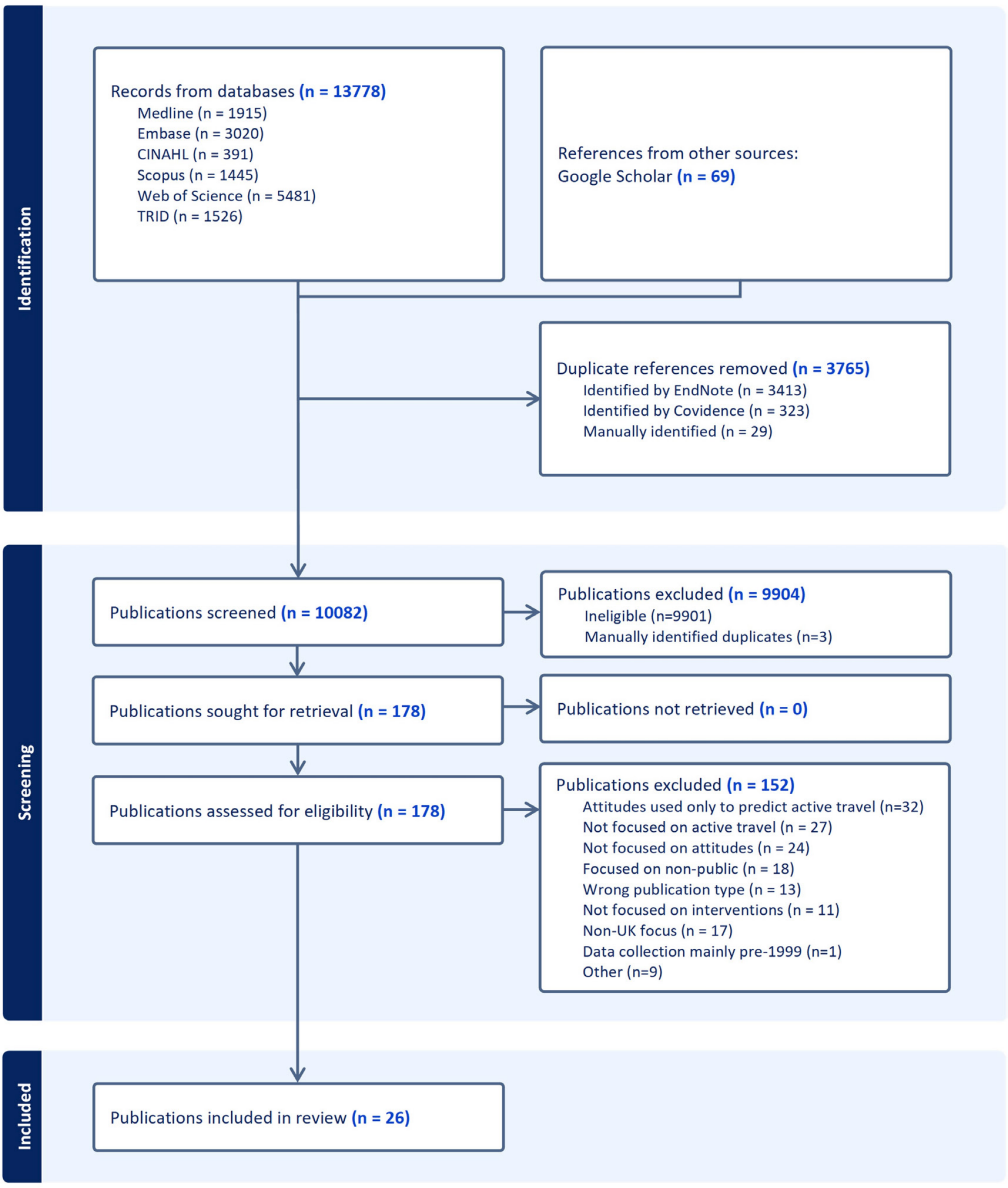
PRISMA flow chart of publication selection process. PRISMA, Preferred Reporting Items for Systematic Reviews and Meta-Analyses.

From 13 847 search results, 26 publications were included (see figure 1). Descriptive statistics of key study features are in table 2 and set out in the publication, intervention and public characteristics sections. Online supplemental file 2 is a more comprehensive table of the characteristics of each included study, stratified by populations of interest affected by each intervention.

**Table 2:** Selected study characteristics

Study characteristics	Categories	N	%
Study approach (n=26)	Qualitative	12	46.2
	Quantitative	6	23.1
	Mixed or multimethods	8	30.8
Geographies (n=29)	UK or Great Britain	3	10.3
	England	3	10.3
	English regions	16	55.2
	Wales	0	0.0
	Scotland	5	17.2
	Northern Ireland	2	6.9
Main population of interest (n=26)	People using street or areas affected	10	38.5
	Residents	4	15.4
	Commuters	3	11.5
	Parents	2	7.7
	Transport professionals	3	11.5
	Samples of public	2	7.7
	Active travellers	1	3.8
	Drivers	1	3.8
Travel modes targeted by interventions (n=26)	Walking and cycling	17	65.4
	Walking	4	15.4
	Cycling	3	11.5
	Cycling and wheeling	1	3.8
	Walking, cycling and wheeling	1	3.8

### Publication characteristics

The search covered 1999 to 2025 but none of the eligible publications was published before 2012. The majority of publications were from 2021 and 2025 (n=14) and the remainder between 2012 and 2019 (n=12). Journal publishers of these works primarily focus on transport (n=15), followed by public health (n=9) and geography and environment (n=2).

Publications took mainly qualitative approaches (n=12), followed by mixed or multimethods (n=8) and quantitative (n=6). Observational study designs were most frequent (n=22) with the remainder experimental or interventional studies (n=3) and one systematic review.

The timing of interventions and studies varied between publications. Prospective or planned interventions (n=4) were interventions that were forthcoming, being tested for feasibility or anticipated. Similarly, some studies analysed attitudes to hypothetical interventions or general descriptions of interventions (n=4). Other publications analysed postimplementation (n=6); preimplementation and postimplementation (n=4); longitudinal with baseline and one follow-up (n=2) and during implementation (n=2). The remaining publications were preintervention only (n=1); combined process and outcome evaluations (n=2) or a systematic review (n=1).

### Intervention characteristics

**Figure 2:**
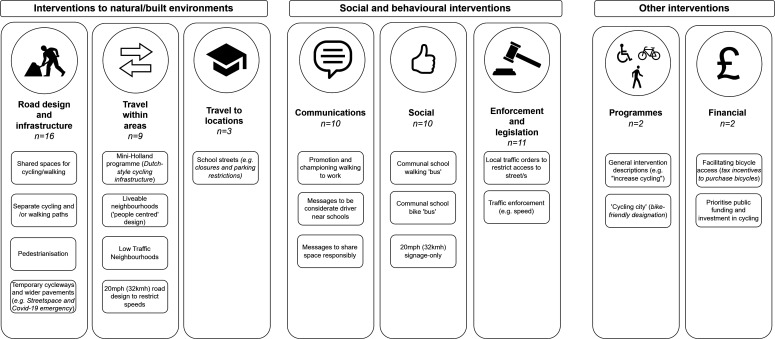
Intervention types included. Figure credits: diagram produced in Draw.io with icons and all images licensed under the CC BY 4.0 (https://jgraph.github.io/drawio/) except UK road sign images (UK Crown copyright, contains public sector information licensed under the Open Government Licence v3.0) and images for ‘Travel to locations’ and ‘Enforcement’ (Wikimedia Commons, used under Creative Commons CC0 1.0 Universal Public Domain Dedication).

Interventions were categorised as changes to natural or built environments; social and behavioural changes; and other interventions (see figure 2 for the intervention categories and specific interventions included). Environmental interventions comprised road design and infrastructure changes (n=16); and facilitating active travel within-area, for example, Low Traffic Neighbourhoods and pedestrianisation (n=9). Social and behavioural interventions included providing incentives (n=10); additional communications activity to encourage or discourage behaviours in favour of active travel (n=10); changes to legislation, policy and/or regulation (n=7) and enforcement penalties (n=4). Active travel to specific locations (n=3) included both environmental changes, such as road closures around schools and encouragements to commuters to travel actively. Other interventions (n=4) were broader programmes, financial interventions or general descriptions of intervention aims.

The majority of publications analysed walking and cycling modes together (n=17). Other mode combinations were analysed in school travel interventions only: cycling and wheeling (n=1) and walking, wheeling and cycling (n=1). All cycling-focused publications (n=3) looked at general or prospective interventions; and walking-focused publications (n=4) looked either at specific interventions or intervention impacts on specific groups, such as older people and on commuters.

Interventions were located in almost every nation or region of the UK with some notable gaps. Interventions in wider political geographies included UK or Great Britain (n=3), England (n=3), Scotland (n=5) and Northern Ireland (n=2) but none specifically in Wales. Publications in specific English regions (n=16) covered all areas except North East England.

Organisations responsible for delivering interventions were mainly local government entities, either single or in ad hoc partnerships (n=18). Other bodies involved were national governments (n=7); transport authorities (n=8); regional governments, combined authorities and metropolitan mayors, including London (n=6); schools (n=2); employers (n=1) and public health services (n=1). ‘Other’ (n=5) includes publications that studied attitudes to a specific intervention but without specific reference to an organisation (n=4); and one intervention led by community members with support from the local authority.

Of the publications reporting postimplementation data collection (n=12), half of the publications concluded data collection within the 12 months after the intervention, or the intervention phase at the time of data collection, had been delivered (n=6). Publications with a longer gap between intervention and data collection mostly undertook single or limited data collection periods at either 12–18 months after (n=2) or more than 19 months after (n=4). Publications in these subgroups that mentioned challenges with scheduling data collection referred to unforeseen changes in intervention timing not matching fixed data collection periods, and funding constraints leading to lost opportunities to collect baseline or follow-up data.

### Public characteristics

Table 2 sets out descriptive statistics of the populations of interest, with details of populations, sample sizes and data collection and intervention dates for each study available in online supplemental file 2. Among the included studies qualitative sample sizes ranged from 7 to 96 (median 30) and quantitative sample sizes from 25 to 4022 (median 413).

### Public attitude themes

This section addresses the research objectives about public attitudes towards active travel interventions in the UK; similarity and variation by place or socioeconomic characteristics and by modes and interventions; and how attitudes have served as a barrier or enabler to active travel policymakers.

Table 3 summarises these findings under three main themes discussed in this section:

- The public generally viewed the concept of active travel positively but perceived it as a niche activity; and attitudes were influenced by people’s prior beliefs, personal characteristics and context.
- Interventions could be unsettling, at least in initial implementation stages, and must be perceived as effective, which was largely judged on personal experience, and perceived as fair to all.
- Attitudes served as a barrier or enabler to policymakers due to the complexities of interventions and the responses to them, resistance to changes that might affect driving, the dominant focus on cycling in discussions about active travel and underlying public cynicism towards governments.

**Table 3:** Themes and findings per results section with frequency of mentions

Section	Theme	Finding	Frequency of mentions in publications
Interventions and how attitudes formed	Active travel positive but niche	Active travel can benefit society	8
		Reduced car traffic, speeds and air pollution would help children actively travel	3
		Active travel is a specialist activity and inconvenient	5
		Cycling is a particularly specialist activity	5
	Prior beliefs, characteristics and context inform attitudes	Prior beliefs inform reasons for support and opposition	3
		Socioeconomic characteristics and travel habits predict intervention support and/or perceived benefit	8
		Little variation in attitudes between intervention areas	1
Interventions can be unsettling and must be effective and fair	Interventions perceived as unsettling, at least initially	Drivers anxious about intervention impacts	6
		Support increases with time but can vary across groups	5
	Must be effective, based on personal experiences	Perceptions of effectiveness based on personal and/or tangible experiences	7
		Interventions can be inconsistent and/or undermined by other policies	5
	Must be fair to all	Interventions should exclude no groups	12
		Interventions should balance incentives and disincentives	2
Attitudes as a barrier or enabler to policymakers	Lack of clear consensus	Public consensus difficult to gauge due to no strong views, even splits or ‘don’t knows’	6
	Driving	Cars are convenient and necessary	4
		Aversion to ‘anti-car’ sentiments and ‘penalties’ for drivers	5
		Specific pro-car/anticycling reaction to infrastructural interventions	3
		Unintended consequences feared, especially towards driving	6
	Walking and wheeling	Lack of walking schemes but appetite for inclusive interventions	4
	Cycling	Cycling is fast, efficient, healthy and growing in popularity	2
		Good infrastructure essential but cyclists feel let down and/or squeezed out by cars	13
		Cycling front of mind when people think of active travel	4
	Public cynicism and poor perceptions	Securing public support is important to policymakers	2
		Public cynicism and poor perceptions of consultation and delivery	7
	Complexity challenges	Complexity of impacts and diverse responses, especially physical changes	8
		Limitations of media for public engagement	3

### Public attitudes towards the interventions and how attitudes were formed

Despite a widespread view that active travel can be positive, considerable sections of the UK public considered it a minority activity. Attitudes were influenced by prior beliefs, people’s characteristics and intervention context.

#### Active travel viewed as generally positive but niche

Publications found people in the UK believed active travel could benefit society through reducing congestion,^54 55^ making streets and communities safer and more pleasant,^56 57^ and it represented a relatively cheap, fast and reliable travel option.^58 59^ Parents perceived walking to school as the ideal mode for children due to health and well-being benefits and incidental social and educational opportunities.^55 60^ Interventions aiming to reduce traffic, vehicle speeds and air pollution were believed to be effective ways to create conditions to encourage children to travel actively.^55 61 62^

While there is evidence some people thought active travel a normal, daily habit^54 63^ it was largely viewed as a specialist activity,^63^ and unsuitable for people with longer commutes or older people.^34^ Logistical challenges or inconvenience put some people off both walking and cycling, including poor weather, needing to carry bulky items, transporting children or dependents, making multistop trips or wearing smart clothes.^58–60^

Cycling was seen as particularly specialist due to being for ‘others’, such as ‘fit, young men’,^58^ requiring confidence and fitness many non-cyclists believed they did not have^54^ or equipment that people did not like to wear, such as helmets.^64^ Cycling was perceived in activist terms with infrastructure perceived as serving a ‘tiny minority of Lycra warriors’^65^ and some interventions, such as communal school bike commutes, thought to ‘send a message’ challenging the status quo of car dominance.^61^

#### Role of prior beliefs, personal characteristics and context

Prior beliefs informed attitudes both to active travel generally and to specific interventions. Perceptions of the urgency and nature of the ‘problem’ shaped support or opposition for interventions with some favouring rapid action to increase active travel due to their views on climate change and public health, while others thought interventions represented rushed, top-down government interference.^66^ Others doubted the nature of the problem as defined by policymakers and therefore whether active travel interventions were necessary^58^ or they believed certain interventions were not appropriate, for example employers should not tell employees how to travel.^59^

The public’s socioeconomic characteristics and travel habits, and how they used the streets in their local environment, predicted support or perceived benefits from interventions.^56 60 67–69^ Contextual differences between areas, such as geographic profiles and existing reliance on cars, could affect support for the same type of intervention.^57^ Linked to this, opinions on cycling interventions correlated with political party preferences^54^ and support for some interventions was associated with particular demographics, for example, support for lower speed limits was higher among older people, women and those who drove shorter total distances.^70^ No studies appeared to deal with how attitudes might vary by ethnicity, and two selected studies mentioned that samples from groups of minoritised ethnicities, lower socioeconomic status groups and faith interests were smaller than desired.^34 58^ The perceived omission of the needs of some members of the population are a potential reason for some public opposition to interventions (see Interventions must be fair to all theme).

There was little evidence that attitudes varied between UK nations and regions but there were some notable gaps in the geographic evidence base, such as rural areas. However, in one publication policymakers cited the attitudes of the public, business and media as a barrier more frequently in London than in other English areas but authors said this was due to the larger number of interventions attempted in the capital.^65^

### Interventions can be unsettling and are judged by effectiveness and fairness

Many people experienced interventions as unsettling changes, particularly in initial stages, and potentially ineffective and unfair.

#### Interventions unsettling initially but more accepted later

Interventions could unsettle people during preintervention phases if they thought they would be potentially affected, particularly those who drive. Drivers’ anxieties included fears about disrupted resident parking from pedestrianisation^68^; anticipated delays, pollution and stress from lower speed limits^57 62^; displaced vehicles from areas with lower traffic^55 56^; or a reduced sense of independence among older people.^71^ Some parents were worried about children walking to school due to cars and other risks, such as ‘stranger danger’ and not wanting to disrupt children’s existing habits.^60^

Some interventions saw support increase and/or opposition decrease postimplementation but not uniformly across social groups.^56 57 68 72 73^ However, as noted earlier, this was mainly based on relatively limited postimplementation data.

#### Personal experiences and observations inform effectiveness perceptions

Effectiveness perceptions were usually based on personal experiences and observations,^34^ for example:

- People saw congestion and speeding cars and concluded that low traffic measures had not worked.^58 71^
- Lack of visible enforcement of lower speed limits led to doubts about effectiveness.^57 62 72^
- Policymakers had greater potential to persuade the public an active travel intervention could work if they used local case studies that seemed comparable to the area affected.^10^

Members of the UK public were less likely than experts to suggest systemic interventions for active travel.^69^ However, the public recognised individual policies cannot solve all transport problems and that combinations of policies are needed,^34^ which included reciprocal or complementary measures such as enforcement against driving offences to accompany low-traffic infrastructure.^71 74^

People could perceive different government policies as inconsistent, for example during the COVID-19 pandemic some people in Low Traffic Neighbourhoods still thought driving the only viable and safe travel mode because government public health messages at the same time asked them to limit public transport use to curtail virus spread.^56^

#### Interventions must be fair to all

Intervention fairness is understood as an important feature of design and delivery.^34 69^ If policymakers use financial penalties they should ‘give something back’^34^ and provide incentives as well as restrictions.^56^

Interventions that redistribute street space or attempt to lower traffic can cause concern that some people and areas will benefit at the expense of others,^58^ for example, due to displacing traffic.^56 74^ Similarly, some prospective behavioural interventions to encourage walking to school could cause concern. Parents thought it would be unfair if families whose children benefited from a communal school walking ‘bus’ did not contribute to the parental volunteer work needed to run the intervention.^60^

Those who disliked active travel interventions commonly mentioned a belief for themselves or on behalf of others that interventions omitted the needs of some groups, such as people who are older,^71^ disabled,^56 58 67^ parents^56 58^ or who rely on a vehicle for work.^58 66^

### How attitudes have been a barrier or facilitator to policymakers

Active travel interventions are often complex and some of the tools policymakers currently use to engage the public involve risks and challenges. Attitudes towards interventions can be difficult to understand, given there is often no apparent consensus among the public alongside strongly vocal representations in favour of driving or cycling.

#### Lack of clear consensus

Despite strong sentiments among drivers and cyclists there is evidence of no strongly expressed views among many in the public. For example, the majority in London were neither in favour of nor against driving or cycling^73^ and the public opposition to prospective speed limiting interventions is milder than support.^70^ Many people simply do not know whether they support or oppose some interventions, either because they were unaware the intervention was taking place or they chose a ‘don’t know’ survey response.^62 68^ In other cases support and opposition are evenly split, which occurred in some Low Traffic Neighbourhoods^56^ and in research gauging if people believe media coverage is fair to cycling or not.^54^

#### Driving

Many drivers perceive cars as convenient and necessary, particularly for groups including parents,^55^ people working or shopping,^63^ disabled people^66^ and older people.^71^ Drivers commonly dislike specific elements of interventions, such as traffic light reprioritisation,^75^ and have a general aversion to ‘anticar’ sentiments or penalties for drivers.^63 76^

Driver opposition can occur at any intervention stage. Many drivers would only support prospective or hypothetical interventions that maintained vehicle priority^10^ and almost half of drivers (49% in 2013) in Britain would oppose any measure perceived to penalise car use.^54^ Car ownership is a predictor of attitudes to cycling and driving and after infrastructure changes to promote cycling in Outer London there was an increase in ‘pro-car’ and ‘anti-cycling’ views.^73 76^

Members of the driving public concerned about active travel frequently cited unintended consequences as a concern. This included increased congestion and car idling^74^; inefficient traffic flows around Low Traffic Neighbourhoods^55^; and increased congestion and journey times as a result of speed limiting interventions.^70^ Similarly, some opposed interventions because of fears of collisions due to driver frustration, more complacent driving and a perceived lack of active traveller responsibility on roads.^62^ Those who disliked cycling interventions cited increased air pollution from cars due to perceiving the interventions caused delays and congestion.^57 76^ Problems with vehicles were often described as ‘driving’ issues whereas cycling problems were personalised as being caused by ‘cyclists’.^76^

#### Cycling

Cyclists viewed cycling as fast, efficient and healthy^63^ and those who cycled regularly were more likely to think there was a ‘cycling boom’ across society.^54^ Good cycling infrastructure interventions were believed to involve separation from cars^64^ and cyclists compared UK exemplars favourably with cycling provision in the Netherlands.^75^ However, cyclists typically felt hostility towards the dominance of the car in urban areas^73^ and often felt let down by poor infrastructure^63 76 77^ which could be ‘tokenistic and precarious’.^77^

The public frequently cited the lack of dedicated infrastructure as a barrier to active travel in general^69^ and believed this undermined the potential of interventions to increase cycling, such as speed limits,^57 62 72^ Low Traffic Neighbourhoods^56^ and school bike buses.^61^

When active travel interventions were combined with public transport the active travel element was less prominently discussed by media.^75^ However, cycling tended to be front of mind in public attitudes about active travel. Improving cycling facilities was the most cited sustainable transport policy among the public and experts^69^ and there was a perception that cycling received disproportionately more funding than other modes, including driving.^65^

#### Walking and wheeling

Among those who walk, there was an appetite for governments to prioritise walking^63^ and for more inclusive practical changes, for example, older people favoured more controlled pedestrian crossings and longer crossing windows.^71^ However, there was a perceived lack of awareness of what a walking promotion intervention would look like and a more general lack of schemes to promote walking.^59^ People who walked reported that infrastructural change, and having to share street space with other active travellers, resulted in anxiety due to a lack of shared understanding about how to use these novel spaces.^77^ As mentioned earlier, wheeling was only specifically analysed in publications about active school travel.

#### Public attitude concerns affect decision-making but the public is largely dissatisfied

Community support for interventions gave confidence to policymakers, who can be risk-averse about public acceptability.^10^ Policymakers perceived public opposition to be a barrier to investment in cycling and adapted to resistance from the public, even anticipating resistance during planning stages.^65^

Ideally, policymakers involved communities throughout the process of designing and delivering an intervention by providing updates, being transparent, encouraging a sense of community ownership for changes, and using good communication through clear terminology and local engagement.^10^ However, publications found policymakers faced public cynicism about their engagement and consultation efforts,^66 68^ including suspected ulterior motives about penalising drivers to raise revenue.^58^ Some policymakers’ decisions were perceived as rushed, lacking consultation or a sense of local knowledge or lacking a guiding strategy.^58 68^ Cynicism could be exacerbated by perceptions that previous interventions had not worked.^59^

#### Complexity of interventions and responses

Publications described how the complexity of interventions led to diverse public responses,^64^ particularly for interventions that changed natural or built environments. Low traffic interventions gave rise to ‘community disharmony’^74^ between residents living within a low traffic area and those living on boundary roads.^56 74^ Specific categories of people affected by pedestrianisation or low traffic initiatives, such as residents or local businesses, had no uniform view and perceived the same intervention effects differently, for example, some residents in low traffic interventions perceived fewer cars as signalling deserted and dangerous streets while others perceived greater safety.^58 68^ Further examples of complexity include an increased belief postintervention that cycling received ‘too much’ support despite improved perceptions of cycling safety^73^; and people who opposed new speed limiting interventions because they anticipated others would ignore the limits.^70^

Media coverage of active travel interventions was more of a concern for policymakers than the public.^34^ Publications found media coverage often treated interventions in a binary way, either positively or negatively. Positive coverage could present idealised versions of interventions that did not match people’s experiences or focused on key moments of interventions, such as the opening of new infrastructure, rather than day-to-day experiences.^75^ Negative coverage could ‘set up or inflame’ conflicts between motoring and cycling interests.^65^

## Discussion

This study synthesised the published evidence about public attitudes to active travel interventions in the UK. The evidence was heterogenous, covering a range of national, regional and local geographic and policy contexts except Wales and North East England; intervention types; and public subgroups, such as residents, commuters and school travellers and related community interests. Publications’ research methods varied but qualitative and observational studies were more common.

While active travel could be positively perceived, it was considered largely niche and exclusive, particularly by the UK majority that did not regularly take part. Interventions to promote active travel, particularly changes to environments, could be perceived as unsettling. The perceived effectiveness and fairness of interventions were important concepts but experienced differently depending on the prior beliefs, travel habits and characteristics of those affected. Attitudinal challenges for policymakers delivering interventions included the inherent complexity of the interventions and responses to them; public cynicism about consultation efforts in a context of long-term decline in trust in governments; and the limitations of media and consultation tools to facilitate common understanding between governments and all groups affected by interventions.

Evidence gaps in publications included a specific focus on modes like walking. Gaps also included studies of interventions in rural areas, and in Wales and North East England, that differ from the UK overall while sharing some distinct commonalities as non-urban and postindustrial areas respectively. While some evidence suggested overall support increased and opposition reduced to interventions after implementation, this was generally based on limited data, either collected soon after implementation or in relatively isolated data collection periods with no sustained collection over longer periods.

Previous reviews of public attitudes to active travel interventions have combined analysis of active travel and other public health interventions^34 35^ or analysed attitudes to specific intervention types,^10^ so this study contributes depth and breadth within the active travel topic across multiple public policy areas and diverse intervention types, travel modes and affected people. However, the exclusion of non-English language studies as well as grey literature, books, theses and conference proceedings was due to resource constraints. While including grey literature can increase relevant and useful sources available, this approach is often resource intensive, can be difficult to report and replicate, and unlike peer-reviewed evidence, these sources have generally not been checked for quality and reporting standards, including competing interests.^78^

The study’s focus on the UK means results cannot necessarily be transferred to other countries. As a scoping review, the study does not assess potential bias, including whether any similarity or variation in public attitudes was due to the quality of measurement, sampling or analysis. Some travel modes and interventions were excluded as non-active, such as e-scooters and fully motorised e-bikes, but these modes have been part of broader transport schemes with active travel interventions and might still have a role in influencing public attitudes towards active travel.

Previous literature has found active travel is viewed as novel, and interventions as unsettling, due to active travel being viewed as inconvenient, risky or unusual especially compared with car use.^79 80^ English-speaking ‘low-cycling countries’^81^ often feature inequalities in cycling participation by age and gender which might add to the perception that cycling is exclusive. Relatively limited postimplementation data are an issue more generally in active travel intervention.^9^ Existing intervention studies also tend to rely on observational, non-experimental designs limiting quantification of the causal effects of interventions on public attitudes.^5^ Though this review focused on the UK, the results are similar to literature from other geographies and across wider transport policy. Cycling interventions have experienced ‘bikelash’ due to various economic, political and practical motivations among the public and politicians^18^; and other studies show the relationships between active travel interventions and attitudes and physical environments are complex.^82^ Established methods for gauging public and community attitudes can struggle to engage the ‘silent majority’ who might favour an intervention,^20^ especially when opposing voices tend to be much more vocal.^83^ The complexity of interventions means that perceptions of the same intervention can vary between social groups and across different contexts.^84^

The findings have implications for policymaking, particularly in the UK. The devolution process of transferring political powers from central government to specific regions and nations is intended to improve the relationship between governments and people, and ensure policymaking is more relevant and acceptable to local people and places.^22^ Yet, these findings show policymakers face a cynical public^85^ and highlight the importance of good communication in all interventions, including those developed and delivered by devolved governments that are more local to the public and communities affected. While growing mistrust of governments is a broader contextual challenge, active travel policymakers should consider how to tackle this and find ways to demonstrate visible and relatable evidence of effectiveness; and ensure intervention processes and outcomes are both fair, ensuring all social groups have access to the opportunity to travel actively,^86^ and are also perceived to be fair. Transport policymaking has been criticised for a ‘decide-announce-defend’ approach to decisions, which involves trying to persuade people affected to agree with technically sound decisions already made; and instead policymakers should use an ‘engage-deliberate-decide’ approach to better value local knowledge and involve people affected at earlier stages of interventions to increase trust and policy legitimacy.^87^ While anticipating and pre-emptively responding to opposition from the public and local communities is not necessarily unhelpful,^65^ interventions are at risk of being less effective if they are weakened at planning or delivery stage^20^ or modified solely to placate opposition.^19^ Where transport literature finds evidence of positive attitude changes postintervention, this usually relies on the intervention as a whole being evaluated as successful,^88^ and in active travel the most effective interventions are multicomponent packages^9^ that change environments and incentivise behaviour change. By contrast, less effective interventions risk being shorter term and less impactful ‘events in systems’ that are later ‘washed out’^89^ of local contexts due to public opposition or being perceived as ineffective or unfair.

This review found evidence gaps that lead to unanswered questions and could motivate future research and policymaking. Some geographies, such as rural areas, were not covered specifically in the included publications which could reflect a shortage of active travel interventions in these contexts. We also found limited evidence on variation of attitudes across ethnic groups. Addressing the unmet demand for walking and wheeling interventions could benefit a much larger section of the population in addition to interventions focused solely on cycling. Given the dominance of qualitative and observational methods included, there could be more experimental or quasi-experimental or natural experimental studies^90^ of interventions to test whether changes are caused by interventions, particularly for interventions that are proven effective but also seem unpopular.^91^ Given recent attempts beyond the UK to remove established active travel interventions,^92^ there is a need to better understand public attitudes in the longer term and gain further insight into how policymakers can sustain support for interventions. over time.

## Conclusions

The review findings suggest planning and implementing interventions can be difficult due to public attitudes and the complexities of interventions and the responses to them. Public engagement is an important part of any potential strategy to tackle low levels of physical activity through active travel. There was a lack of policy-relevant evidence about the practical strategies needed to address public engagement challenges, particularly how to address public cynicism and lack of trust in government and how to practically ensure people affected by interventions perceive them as effective and fair. More research is needed into the causal effect of interventions and on attitudes over time. Despite these gaps, this scoping review provides insight for policymakers who would like to develop and deliver effective active travel strategies for increasing physical activity across populations with public and community support.

## Supplementary material

10.1136/bmjopen-2025-115598 online supplemental file 1

10.1136/bmjopen-2025-115598 online supplemental file 2

## Data Availability

This review draws on published research only. No new data were collected or generated in undertaking this study.
