# Supplementary material for: Scoping review of public attitudes to government active travel interventions in the UK
Source: BMJ Open. 2026 Aug 14;16(8):e115598. doi: 10.1136/bmjopen-2025-115598 (PMC13479531; doi:10.1136/bmjopen-2025-115598)
Supplement: Supplementary file 1 [file bmjopen-2025-115598.reviewer_comments.pdf]

PEER REVIEW HISTORY

BMJ Open publishes all reviews undertaken for accepted manuscripts. Reviewers are asked to complete a checklist review form and are provided with free text boxes to elaborate on their assessment. These free text comments are reproduced below.

ARTICLE DETAILS

Title (Provisional)

Scoping review of public attitudes to government active travel interventions in the UK

Authors

Morris, Nick; Jago, Russell (Russ); Russell, Helen P; Salway, Ruth E; Morgan, Kelly; Kipping, Ruth

---

VERSION 1 - REVIEW

**Reviewer**	**1**
**Name**	**Andriyani, Fitria**
**Affiliation**	**Universitas Negeri Yogyakarta, Department of Sports Education**
**Date**	**24-Feb-2026**
**COI**	**None**

This scoping review aims to synthesise evidence on public attitudes toward active travel interventions in the UK. Overall, the background is sufficiently developed, the search strategy appears comprehensive, and the inclusion of the PRISMA-ScR checklist strengthens the methodological transparency. The criteria for identifying relevant publications are clearly described.

However, several methodological aspects require clarification.

Exclusion Criteria

The rationale for excluding grey literature, student theses, and conference proceedings requires further justification. In the context of a scoping review—where the aim is typically to map the breadth of available evidence—such sources can provide valuable insights and reduce publication bias.

Screening Process and Reviewer Agreement

The manuscript states that two reviewers independently screened 30% of randomly selected titles and abstracts, resolved conflicts, and then one reviewer screened the remaining 70%. The rationale for this decision needs clarification.

Risk of Bias in Study Selection

Given that a substantial proportion of studies were screened by a single reviewer, the authors should clarify how potential selection bias was minimised.

Study Limitations

The manuscript would benefit from a more explicit and critical discussion of its limitations. For instance, the authors should acknowledge potential limitations from excluding grey literature.

Reviewer 2

Name Zhandire, Tracy

Affiliation University of KwaZulu-Natal College of Health Sciences, School of Nursing & Public Health

Date 12-Mar-2026

COI None

		**Comment**
		I would like to commend the authors for addressing a timely and important topic. A scoping review of public attitudes towards government active travel interventions fills a meaningful gap in the literature and has clear relevance for policymakers across the UK's devolved nations. This is a valuable contribution to the field.
1	Abstract	The abstract is generally well structured and clearly presents the objective, methods, and key findings of the study. The rationale for examining public attitudes towards active travel interventions in the UK is also well articulated. However, several areas could be strengthened.<br>First, the Results section of the abstract could be improved by presenting the key findings more succinctly and clearly highlighting the main themes that emerged from the review. For example, summarising the principal factors influencing public

		attitudes (e.g., perceptions of effectiveness, fairness, and travel habits) in a more concise manner would improve readability.<br>Second, the abstract does not report the total number of sources included in the review (n=26) or the time period covered (1999 to present). Reporting these details is recommended under PRISMA-ScR guidance (Item 2) and would help readers quickly assess the scope and scale of the evidence base.<br>Finally, the abstract would benefit from a brief concluding sentence that emphasises the implications of the findings for policymakers or future research, providing the reader with a clearer sense of the review's contribution and practical relevance.
2	Introduction<br>Page 3, line 7 – 10, "Populations globally are at risk of health problems… due to low levels of physical activity."	The opening effectively establishes the public health importance of physical activity; however, the transition from global physical inactivity to the specific focus on active travel in the UK could be made more explicit to strengthen the contextual framing of the study. This flow could help, Global problem → Active travel → UK policy context → Research gap.
3	Page 3, line 15 - 16<br>"Active travel… is routine physical activity through walking, wheeling and cycling for everyday purposes."	The definition of active travel is clearly provided. However, the introduction could be strengthened by briefly explaining why active travel is particularly important as a public health strategy. While the role of active travel in increasing physical activity is mentioned, highlighting additional co-benefits, such as environmental sustainability, reduced traffic congestion, improved air quality, and broader population health benefits, would provide stronger context for its policy relevance.<br>For example, the authors could consider adding a sentence such as:

		In addition to increasing physical activity, active travel has been associated with broader co-benefits, including reductions in air pollution and traffic congestion, improvements in urban mobility, and potential gains for population health and environmental sustainability.
**4**	Page 3, line 21 -24,<br><br>“In recent decades the process of devolution… has involved the United Kingdom holding referenda…”	The discussion of devolution provides important policy context. However, the link between devolution and active travel policy variation could be clarified more explicitly to demonstrate how devolved governance structures shape the policy environment relevant to the review.
5	Page 3, line 33-37<br><br>“However, audit bodies report that all four governments are falling short of their objectives…”	The sentence highlights an important policy challenge; however, the connection between these policy shortfalls and the relevance of examining public attitudes could be made more explicit. Clarifying how public acceptance may influence the implementation and sustainability of active travel interventions would strengthen the justification for the review.<br>For example, the authors could consider adding a linking sentence such as:<br>One factor that may contribute to these challenges is the level of public acceptance of active travel interventions, as public support can influence whether such policies are successfully implemented, sustained, or modified during planning and delivery stages.
6	Page 3, line 42 – 45<br><br>“As well as being effective, public health interventions must also be deliverable which includes being acceptable to the public…”	The introduction identifies public acceptability as a key concept; however, the concept is not clearly defined. Providing a brief clarification of what is meant by “acceptability” in the context of public health or policy implementation would strengthen the conceptual framing of the study.<br>For example, the authors could briefly clarify the concept by adding a sentence such as:

		"Public acceptability refers to the extent to which individuals or communities support, tolerate, or oppose a policy or intervention, which can influence its implementation, sustainability, and overall effectiveness."
7	Page 3, line 57 - 60<br>"Existing reviews into the acceptability of public health interventions have combined active travel with other public health policy areas…"	This is a literature gap clearly identified; however, the argument could be strengthened by briefly summarising the limitations of previous reviews to demonstrate why a dedicated scoping review focused specifically on active travel interventions is needed.<br>For example, the authors could clarify the limitations of prior work by adding a sentence such as:<br>Previous reviews have examined the acceptability of public health interventions more broadly or have focused on specific types of active travel measures, which may limit understanding of public attitudes across the full range of active travel interventions.
**8**	Research question clarity<br>"The primary aim of this scoping review was to identify the evidence on public attitudes towards government active travel interventions…"	The aim of the review is clearly stated; however, the manuscript does not explicitly present the research question. For clarity and alignment with scoping review reporting practices, the authors could consider clearly stating the research question in addition to the aim.<br>For example, the authors could present a question such as:<br>What evidence exists on public attitudes towards government active travel interventions across the devolved nations and regions of the UK?
9	"The scoping review method was particularly appropriate for this topic given the	The rationale for using a scoping review is appropriate; however, the justification could be strengthened by briefly clarifying what types of evidence diversity are expected in this field. Providing examples of the

	potential diversity of evidence…”	different forms of evidence (e.g., policy analyses, public opinion surveys, qualitative studies, or mixed-methods research) would help the reader better understand why a scoping review approach is suitable for this topic.<br>For example, the authors could clarify this by adding a sentence such as:<br>“Evidence on public attitudes towards active travel interventions may include a range of study designs, such as policy analyses, public opinion surveys, qualitative studies exploring stakeholder perspectives, and mixed-methods research examining implementation experiences.”
10	“The secondary aims were to: summarise the evidence and data on public attitudes…”	The secondary aims are comprehensive; however, the sentence is quite long and includes multiple objectives, which may affect readability. Consider presenting the objectives as a clearer list or separating them into shorter sentences to improve clarity and highlight the key analytical goals of the review.<br>For example, the authors could present the objectives as separate bullet points or numbered statements, such as:<br>To summarise the evidence on public attitudes towards active travel interventions in the UK since the advent of devolution in 1999.<br>1. To identify themes in public support and opposition to active travel interventions.<br>2. To explore similarities and differences between UK nations, regions, and socio-economic groups.

		3. To examine how public attitudes may act as barriers or enablers to policymakers implementing active travel interventions.
11		The introduction contains relevant background information and clearly highlights the public health relevance of active travel and the importance of understanding public attitudes towards related interventions. However, the section would benefit from clearer structuring and stronger transitions between key ideas. At present, several concepts, including the global burden of physical inactivity, the policy context of devolution, and the role of public acceptability, are introduced in close succession, which makes the overall argument somewhat difficult to follow. Strengthening the links between these elements and more explicitly connecting the policy context, literature gap, and research objectives would improve the logical flow and readability of the introduction.
12	Methodology	The methodology is generally well structured and appropriately follows established scoping review frameworks, including the PRISMA-ScR guidance, the Joanna Briggs Institute manual, and Arksey and O'Malley's five-stage process. The description of the search strategy, screening process, and data charting procedures provides useful transparency.<br>However, several areas could be clarified to further strengthen methodological transparency. First, while the involvement of stakeholders in Stage 1 is mentioned, the description lacks sufficient detail to assess how this process was conducted. Specifically, it is unclear how stakeholders were identified and selected, how many were consulted, what method was used to gather their input, and how their contributions

		concretely influenced the final research question or scope of the review. Providing this detail would improve transparency and align with best practice for scoping reviews that incorporate stakeholder engagement. Second, while the search strategy and databases are clearly listed, it may be helpful to briefly clarify whether any restrictions were applied (e.g., language or publication type) within the search stage itself, as these are currently described primarily in the eligibility criteria.<br>Third, the manuscript does not clearly describe whether data charting was conducted independently by more than one reviewer or whether a consensus process was used to resolve disagreements. PRISMA-ScR Item 10 requires authors to describe whether charting was done independently or in duplicate, and to outline any processes for confirming data with investigators. Finally, the section describing data synthesis could provide greater detail on how the thematic analysis was conducted and how themes relating to public attitudes were identified, which would further support reproducibility.<br>Overall, the methodology is clearly presented and follows recognised scoping review procedures, but these clarifications would meaningfully improve transparency and reproducibility.
13		The manuscript lists the databases searched and provides a broad description of the search strategy; however, it is not entirely clear whether the full electronic search strings are available in sufficient detail to allow replication. PRISMA-ScR Item 8 requires that the full search strategy for at least one database be presented, including any limits applied. If

		the complete search strings are included in supplementary materials, a clearer signpost to this within the methods section would improve transparency. If they are not currently provided in full, the authors should consider adding them.
14	Table 1 (inclusion and exclusion criteria)	Table 1 clearly outlines the inclusion and exclusion criteria used to identify relevant publications. However, some sections — particularly under Concept 2 (public attitudes to active travel interventions) — contain detailed descriptions that make the table somewhat difficult to follow. Consider simplifying or separating the criteria for public attitudes and active travel interventions into clearer bullet points or subcategories to improve readability.
15	Results section	The Results section provides detailed descriptive information about publication characteristics, study designs, and intervention types, which is helpful for understanding the scope of the evidence base. However, given that the primary aim of the review is to synthesise public attitudes toward active travel interventions, the section may benefit from placing greater emphasis on the thematic findings related to public attitudes.<br>The themes presented in the section 'Public attitude themes, such as active travel being viewed as positive but niche, the influence of prior beliefs, and how socio-economic characteristics shape support or opposition, appear central to the review's objectives. Bringing these thematic insights more prominently into the Results section, or summarising them earlier, could strengthen the alignment between the findings and the stated aims of the review.

16		The tables and figures (e.g., Table 2 and Table 3) provide detailed information; however, some of the information presented in the text appears to repeat what is already shown in the tables. Consider reducing some textual repetition and referring readers to the tables more directly to improve conciseness.
17		The section describing public attitude themes is informative, but the themes could be more clearly summarised at the beginning of the subsection before presenting detailed examples. A brief introductory statement outlining the key themes may help guide the reader through the results.
18		In some parts of the Results section, terms such as public attitudes, perceptions, beliefs, and opinions appear to be used interchangeably. Clarifying whether these terms are used synonymously or represent different constructs could improve conceptual clarity.
19		Although examining similarities and differences across socio-economic groups is listed as a secondary aim, the review does not appear to systematically address how public attitudes may differ by ethnicity, disability status, or other dimensions of social inequality beyond socio-economic position. Given that active travel interventions can have differential impacts across population groups, a brief reflection on what the evidence does or does not tell us about equity — and acknowledgement of this as a limitation where evidence is absent — would strengthen the review's contribution and its relevance for equitable policymaking.
20	Discussion interpretation	The Discussion effectively summarises the key findings and situates them within the broader literature

		on active travel and public attitudes. The comparison with previous reviews and international literature is particularly helpful in contextualising the findings.
21	Link between results and policy implications	While the discussion includes important reflections on policymaking and public engagement, the connection between the review findings and the proposed policy implications could be articulated more explicitly. Clarifying how the evidence directly informs these recommendations would strengthen the argument.
22	Limitations of the review	The section appropriately acknowledges several limitations, including the lack of bias assessment and the UK-specific focus. However, the discussion could also briefly reflect on potential publication bias or language restrictions, given that only English-language studies were included.
23		The identification of research gaps—such as the limited evidence on rural contexts and longer-term evaluations—is valuable. This section could be strengthened by more clearly highlighting specific priorities for future research, particularly regarding longitudinal studies and causal evaluations of interventions.
24	Conclusion	The conclusion provides a clear summary of the key findings and emphasises the role of public attitudes in shaping the implementation of active travel interventions. The section is concise and appropriately aligned with the results of the review.
25		The conclusion provides a concise summary of the study; however, it could be strengthened by more clearly reiterating the key insight of the review. In particular, emphasising the complexity of public attitudes toward active travel interventions and the

		importance of effective public engagement in policymaking would help reinforce the central contribution of the study and highlight its relevance for policy and practice.
26		The call for further research is appropriate; however, the conclusion may benefit from briefly highlighting practical implications for policymakers, particularly regarding communication strategies, stakeholder engagement, and long-term evaluation of interventions.
27		While the UK-specific focus of the review is acknowledged in the limitations section, the conclusion does not reflect on what this means for the applicability of the findings beyond the UK context. Given that active travel policy is an area of growing international interest, briefly acknowledging in the conclusion that findings may not be directly transferable to other national or policy contexts, due to differences in governance structures, built environments, and cultural attitudes, would provide a more balanced and appropriately cautious closing statement
28	References	The reference list is relevant, wide-ranging, and appropriately includes policy reports and government documents alongside academic literature, which is well-suited to the policy-oriented nature of this scoping review. However, some inconsistencies in formatting are noted across the list — for example, variation in the inclusion of issue numbers, page ranges, and DOIs, inconsistent presentation of journal titles (some abbreviated, others written in full), and occasional use of URLs where DOIs are available. A final check against BMJ Open's referencing requirements to ensure consistency across all citations would strengthen the

		presentation of an otherwise comprehensive and well-constructed reference list.

VERSION 1 - AUTHOR RESPONSE

Reviewer #1

Reviewer #1 general comments to the authors
This scoping review aims to synthesise evidence on public attitudes toward active travel interventions in the UK. Overall, the background is sufficiently developed, the search strategy appears comprehensive, and the inclusion of the PRISMA-ScR checklist strengthens the methodological transparency. The criteria for identifying relevant publications are clearly described. However, several methodological aspects require clarification.

Response: Thanks to the reviewer for the positive remarks about the fundamental aspects of the manuscript, and for requests for clarification that we respond to below.

Reviewer 1, Comment 1 (Exclusion Criteria)
The rationale for excluding grey literature, student theses, and conference proceedings requires further justification. In the context of a scoping review—where the aim is typically to map the breadth of available evidence—such sources can provide valuable insights and reduce publication bias.

Response: We have added justification for this decision to the Strengths/Limitations summary content for the article front page (lines 67-68). Briefly, we sought to make the search scope as broad and comprehensive as possible while being feasible in light of resource constraints. As mentioned in response to Reviewer 1 Comment 4, we have added this point as a limitation in the Discussion section (lines 530-533).

Reviewer 1, Comment 2 (Screening Process and Reviewer Agreement)
The manuscript states that two reviewers independently screened 30% of randomly selected titles and abstracts, resolved conflicts, and then one reviewer screened the remaining 70%. The rationale for this decision needs clarification.

Response: Similarly to Comment 1, our approach sought to make the search broad and comprehensive enough for the research question but also feasible. The proportion of independently screened titles and abstracts exceeds the 20-25% proportion used in other published scoping reviews in public health [1, 2]. Our choice of a 30% proportion

for independent screening draws on methods literature, for example Cochrane guidance on rapid reviews recommends at least 20% independent screening with a calibration or conflict resolution stage [3], and we increased this to 30% as this was a scoping review design. The original manuscript prominently reports this screening approach in the Strengths/Limitations summary content for the article front page (lines 60-61) but we have now added a reference to the methods literature in our methods section (lines 175-176 and 180-181). Please also see Comment 3 for the steps we took to reduce risk of bias with this approach.

Reviewer 1, Comment 3 (Risk of Bias in Study Selection)
Given that a substantial proportion of studies were screened by a single reviewer, the authors should clarify how potential selection bias was minimised.

Response: *The original screening criteria were pre-specified and outlined in the publicly available study protocol (https://osf.io/58a9x/files/8zpxn). Moreover, criteria were calibrated by the two reviewers assessing 30% of randomly selected papers from the search. There was high proportionate agreement between the reviewers for inclusion and exclusion decisions, exceeding the recommended threshold for agreement specified in scoping review literature when independently screening a proportion of full search results [4] (referred to in line 176). Text outlining these changes has been added to the methods section (please see lines 172-181)*

Reviewer 1, Comment 4 (Study Limitations)
The manuscript would benefit from a more explicit and critical discussion of its limitations. For instance, the authors should acknowledge potential limitations from excluding grey literature.

Response: Please also see response to Reviewer 1, Comment 1. We have now acknowledged this limitation and its potential implications within the Discussion section (lines 530-533).

Reviewer #2

Reviewer #2 comments to the author

I would like to commend the authors for addressing a timely and important topic. A scoping review of public attitudes towards government active travel interventions fills a meaningful gap in the literature and has clear relevance for policymakers across the UK's devolved nations. This is a valuable contribution to the field.

The abstract is generally well structured and clearly presents the objective, methods, and key findings of the study. The rationale for examining public attitudes towards active travel interventions in the UK is also well articulated. However, several areas could be strengthened.

Response: Thanks to the reviewer for the kind words about the contribution of this manuscript and abstract, and for the comments to help improve it.

Reviewer 2, Comment 1a (Abstract)

First, the Results section of the abstract could be improved by presenting the key findings more succinctly and clearly highlighting the main themes that emerged from the review. For example, summarising the principal factors influencing public attitudes (e.g., perceptions of effectiveness, fairness, and travel habits) in a more concise manner would improve readability

Response: In line with your suggestions, we have reworded this section (lines 37-38) to aid the reader.

Reviewer 2, Comment 1b (Abstract)

Second, the abstract does not report the total number of sources included in the review (n=26) or the time period covered (1999 to present). Reporting these details is recommended under PRISMA-ScR guidance (Item 2).

Response: Details can now be found at the start of the Results section in the abstract (line 31).

Reviewer 2, Comment 1c (Abstract)
Finally, the abstract would benefit from a brief concluding sentence that emphasises the implications of the findings for policymakers or future research, providing the reader with a clearer sense of the review's contribution and practical relevance.

Response: We have added a concluding sentence (lines 47-49) to emphasise the review's contribution and relevance to policymaking.

Reviewer 2, Comment 2 (Introduction)

The opening effectively establishes the public health importance of physical activity; however, the transition from global physical inactivity to the specific focus on active travel in the UK could be made more explicit to strengthen the contextual framing of the study. This flow could help, Global problem → Active travel → UK policy context → Research gap.

Response: We understand this to be similar to Comment 11, which recommends improving the transitions between concepts in the Introduction. In response we have changed the ordering of the introduction paragraphs to reflect the logical flow that the reviewer suggested (this paragraph has been moved and is now found on lines 97-113). Within this text we added an additional line, drawn from our study protocol, to further justify the relevance of the UK active travel policy context to public attitudes and acceptability of interventions (lines 102-103).

Reviewer 2, comment 3 (Introduction: "Active travel… is routine physical activity through walking, wheeling and cycling for everyday purposes.")

The definition of active travel is clearly provided. However, the introduction could be strengthened by briefly explaining why active travel is particularly important as a public health strategy. While the role of active travel in increasing physical activity is mentioned, highlighting additional co-benefits, such as environmental sustainability, reduced traffic congestion, improved air quality, and broader population health benefits, would provide stronger context for its policy relevance. For example, the authors could consider adding a sentence such as: In addition to increasing physical activity, active travel has been associated with broader co-benefits, including reductions in air pollution and traffic congestion, improvements in urban mobility, and potential gains for population health and environmental sustainability.

Response: We have added a sentence and accompanying citation to the introduction to reflect these co-benefits (lines 81-84).

Reviewer 2, Comment 4 (Introduction: "In recent decades the process of devolution…")

The discussion of devolution provides important policy context. However, the link between devolution and active travel policy variation could be clarified more explicitly to demonstrate how devolved governance structures shape the policy environment relevant to the review.

Response: While the manuscript set outs how devolution can lead to variation in policy approaches we have added a recent example from Wales (2023) of a large-scale active travel intervention implemented directly as a result of powers newly devolved from central government in 2018 (lines 104-107).

Reviewer 2, Comment 5 (Introduction: "However, audit bodies report that all four governments are falling short of their objectives…")

The sentence highlights an important policy challenge; however, the connection between these policy shortfalls and the relevance of examining public attitudes could be made more explicit. Clarifying how public acceptance may influence the implementation and sustainability of active travel interventions would strengthen the justification for the review. For example, the authors could consider adding a linking sentence such as: "One factor that may contribute to these challenges is the level of public acceptance of active travel interventions, as public support can influence whether such policies are successfully implemented, sustained, or modified during planning and delivery stages."

Response: Please see response to Comment 6.

Reviewer 2, Comment 6 (Introduction: "As well as being effective, public health interventions must also be deliverable...")

The introduction identifies public acceptability as a key concept; however, the concept is not clearly defined. Providing a brief clarification of what is meant by "acceptability" in the context of public health or policy implementation would strengthen the conceptual framing of the study. For example, the authors could briefly clarify the concept by adding a sentence such as: "Public acceptability refers to the extent to which individuals or communities support, tolerate, or oppose a policy or intervention, which can influence its implementation, sustainability, and overall effectiveness."

Response: We have added a cited definition in the public health context of public acceptability (lines 90-91), which also reflects the inclusion criteria in Table 1 (See inclusion criteria for Concept 2a). This revision is followed by the existing text, which sets out how public attitudes can help or hinder policymaking (lines 91-96) and therefore now makes clearer why public acceptance matters to policy implementation (also see Reviewer 2, Comment 5).

Reviewer 2, Comment 7 (Introduction: "Existing reviews into the acceptability of public health interventions")

This is a literature gap clearly identified; however, the argument could be strengthened by briefly summarising the limitations of previous reviews to demonstrate why a dedicated scoping review focused specifically on active travel interventions is needed. For example, the authors could clarify the limitations of prior work by adding a sentence such as: Previous reviews have examined the acceptability of public health interventions more broadly or have focused on specific types of active travel measures, which may limit understanding of public attitudes across the full range of active travel interventions.

Response: As suggested, we have added a point (lines 118-119) about the limitations of existing reviews to further emphasise the need for this scoping review.

Reviewer 2, Comment 8 (Introduction: Research question clarity)

The aim of the review is clearly stated; however, the manuscript does not explicitly present the research question. For clarity and alignment with scoping review reporting practices, the authors could consider clearly stating the research question in addition to the aim. For example, the authors could present a question such as: What evidence exists on public attitudes towards government active travel interventions across the devolved nations and regions of the UK?

Response: To aid the reader, we have revised the wording so the research question is now presented in question form (lines 127-129) consistent with the phrasing originally specified in the study protocol.

Reviewer 2, Comment 9 (Introduction: "The scoping review method was particularly appropriate for this topic…")

The rationale for using a scoping review is appropriate; however, the justification could be strengthened by briefly clarifying what types of evidence diversity are expected in this field. Providing examples of the different forms of evidence (e.g., policy analyses, public opinion surveys, qualitative studies, or mixed-methods research) would help the reader better understand why a scoping review approach is suitable for this topic. For example, the authors could clarify this by adding a sentence such as: "Evidence on public attitudes towards active travel interventions may include a range of study designs, such as policy analyses, public opinion surveys, qualitative studies exploring stakeholder perspectives, and mixed-methods research examining implementation experiences."

Response: We revised the manuscript to this effect (lines 132-134), noting the anticipated evidence diversity that made a scoping review approach particularly suitable.

Reviewer 2, Comment 10 (Introduction: "The secondary aims were to…")

The secondary aims are comprehensive; however, the sentence is quite long and includes multiple objectives, which may affect readability. Consider presenting the objectives as a clearer list or separating them into shorter sentences to improve clarity and highlight the key analytical goals of the review. For example…

Response: This part has been formatted accordingly into a list form (lines 136-145).

Reviewer 2, Comment 11 (Introduction)

The introduction contains relevant background information and clearly highlights the public health relevance of active travel and the importance of understanding public attitudes towards related interventions. However, the section would benefit from clearer structuring and stronger transitions between key ideas. At present, several concepts, including the global burden of physical inactivity, the policy context of devolution, and the role of public acceptability, are introduced in close succession, which makes the overall argument somewhat difficult to follow. Strengthening the links between these elements and more explicitly connecting the policy context, literature gap, and research objectives would improve the logical flow and readability of the introduction.

Response: Please see response and revisions in Reviewer 2, Comment 2 which also recommended improving the Introduction's use of concepts and logical flow.

Reviewer 2, Comment 12a (Methodology) The methodology is generally well structured and appropriately follows established scoping review frameworks, including the PRISMA-ScR guidance, the Joanna Briggs Institute manual, and Arksey and O'Malley's five-stage process. The description of the search strategy, screening process, and data charting procedures provides useful transparency. However, several areas could be clarified to further strengthen methodological transparency.

First, while the involvement of stakeholders in Stage 1 is mentioned, the description lacks sufficient detail to assess how this process was conducted. Specifically, it is unclear how stakeholders were identified and selected, how many were consulted, what method was used to gather their input, and how their contributions concretely influenced the final research question or scope of the review. Providing this detail would improve transparency and align with best practice for scoping reviews that incorporate stakeholder engagement.

Response: Full details of the consultation process are located in the study protocol however, we have revised the manuscript (lines 156-160) to add further details about the consultation process and how we improved the study design in response to consultee feedback.

Reviewer 2, Comment 12b (Methodology)

Second, while the search strategy and databases are clearly listed, it may be helpful to briefly clarify whether any restrictions were applied (e.g., language or publication type) within the search stage itself, as these are currently described primarily in the eligibility criteria.

Response: We have added brief mention in the main text (lines 162-3) that the search restrictions are outlined in Table 1 and more fully in the study protocol. The specific search syntax we used, including limits applied, are fully available in the Medline search strategy shared in Supplementary File 1 (referred to in line 166).

Reviewer 2, Comment 12c (Methodology)

Third, the manuscript does not clearly describe whether data charting was conducted independently by more than one reviewer or whether a consensus process was used to resolve disagreements. PRISMA-ScR Item 10 requires authors to describe whether charting was done independently or in duplicate, and to outline any processes for confirming data with investigators.

Response: We have added a brief reference (lines 190-191) to make this aspect of the charting process more explicit.

Reviewer 2, Comment 12d (Methodology)

Finally, the section describing data synthesis could provide greater detail on how the thematic analysis was conducted and how themes relating to public attitudes were identified, which would further support reproducibility.

Response: We have added content (lines 192-195) setting out the thematic analysis process used.

Reviewer 2, Comment 12e (Methodology)

Overall, the methodology is clearly presented and follows recognised scoping review procedures, but these clarifications would meaningfully improve transparency and reproducibility.

Response: Thank for your positive comments about the methodology section in general.

Reviewer 2, Comment 13

The manuscript lists the databases searched and provides a broad description of the search strategy; however, it is not entirely clear whether the full electronic search strings are available in sufficient detail to allow replication. PRISMA-ScR Item 8 requires that the full search strategy for at least one database be presented, including any limits applied. If the complete search strings are included in supplementary materials, a clearer signpost to this within the methods section would improve transparency. If they are not currently provided in full, the authors should consider adding them.

Response: The first reference to the Medline search strategy specifies (line 166) that this is in Supplementary File 1. This strategy includes the complete search syntax and limits applied (which was also requested in Reviewer 2's Comment 12b).

Reviewer 2, Comment 14 (Table 1 inclusion and exclusion criteria)

Table 1 clearly outlines the inclusion and exclusion criteria used to identify relevant publications. However, some sections — particularly under Concept 2 (public attitudes to active travel interventions) — contain detailed descriptions that make the table somewhat difficult to follow. Consider simplifying or separating the criteria for public attitudes and active travel interventions into clearer bullet points or subcategories to improve readability.

Response: The BMJ Open editorial team also requested a similar revision separately. To aid the reader we have shortened the table to include the key criteria and signpost readers to the study protocol (line 163) for the full rationale for the criteria.

Reviewer 2, Comment 15 (Results)

The Results section provides detailed descriptive information about publication characteristics, study designs, and intervention types, which is helpful for understanding the scope of the evidence base. However, given that the primary aim of the review is to synthesise public attitudes toward active travel interventions, the section may benefit from placing greater emphasis on the thematic findings related to public attitudes. The themes presented in the section 'Public attitude themes, such as active travel being viewed as positive but niche, the influence of prior beliefs, and how socio-economic characteristics shape support or opposition, appear central to the review's objectives. Bringing these thematic insights more prominently into the Results section, or summarising them earlier, could strengthen the alignment between the findings and the stated aims of the review.

Response: Thanks for the positive comments about the Results section.

Please see our responses to Comments 16 and 17 which relate to this comment about giving due prominence to the thematic findings while minimising repetition between tables and the main manuscript text.

Reviewer 2, Comment 16

The tables and figures (e.g., Table 2 and Table 3) provide detailed information; however, some of the information presented in the text appears to repeat what is already shown in the tables. Consider reducing some textual repetition and referring readers to the tables more directly to improve conciseness.

Response: We have carefully reviewed these tables, and the manuscript main body, to check for textual repetition. The BMJ Open editorial team also requested that Table 2 be shortened or moved to supplementary materials. In response we have transferred the originally submitted Table 2 to Supplementary File 2 as it contains materials required in the PRISMA-ScR checklist. The revised Table 2 is now a very brief summary table of key characteristics of included studies.

We believe Table 3 does not need further revision. The table does repeat the headline findings and themes in brief but this is intended to help readers navigate the long-form thematic descriptions and understand the fit and hierarchy of the themes and findings as a whole (see related responses to Comments 15 and 17).

Reviewer 2, Comment 17

The section describing public attitude themes is informative, but the themes could be more clearly summarised at the beginning of the subsection before presenting detailed examples. A brief introductory statement outlining the key themes may help guide the reader through the results.

Response: We have added three brief descriptions to the start of this section (lines 284-293) which summarise key features of the three main themes and main findings in each.

Reviewer 2, Comment 18

In some parts of the Results section, terms such as public attitudes, perceptions, beliefs, and opinions appear to be used interchangeably. Clarifying whether these terms are used synonymously or represent different constructs could improve conceptual clarity.

Response: Yes, we use these terms interchangeably in Results to reflect the inclusion criteria (as set out in Table 1, Concept 2a).

Reviewer 2,Comment 19

Although examining similarities and differences across socio-economic groups is listed as a secondary aim, the review does not appear to systematically address how public attitudes may differ by ethnicity, disability status, or other dimensions of social inequality beyond socio-economic position. Given that active travel interventions can have differential impacts across population groups, a brief reflection on what the evidence does or does not tell us about equity — and acknowledgement of this as a limitation where evidence is absent — would strengthen the review's contribution and its relevance for equitable policymaking.

Response: We have added content (lines 338-341) stating that the selected literature does not deal in depth with potential variation in attitudes by ethnicity. We have also added references (see line 341) to two of the included papers that briefly mention limitations in their sampling approach in this area, namely that samples were either smaller than desired or non-existent for minoritised ethnic groups, lower socio-economic status groups, and faith interests.

Findings about public attitudes that relate to equity are split between two themes but we believe this is appropriate. The first theme presents how public views can vary by some characteristics (lines 331-338); and the second theme covers how the interests of some groups in society, such as older and/or disabled people, constitute a reason for some opposition to interventions (lines 396-399). We have added a mention of this split (line 343) in the first theme to ensure readers are aware.

Reviewer 2, Comment 19 (Discussion interpretation)

The Discussion effectively summarises the key findings and situates them within the broader literature on active travel and public attitudes. The comparison with previous reviews and international literature is particularly helpful in contextualising the findings.

Response: Thank you for these positive comments about the Discussion section.

Reviewer 2, Comment 21 (Link between results and policy implications)

While the discussion includes important reflections on policymaking and public engagement, the connection between the review findings and the proposed policy implications could be articulated more explicitly. Clarifying how the evidence directly informs these recommendations would strengthen the argument.

Response: We have made a series of small revisions to make more explicit the connection between the review's findings and the policymaking reflections (see lines 559-563, 566-570, and 577-578).

Reviewer 2, Comment 22 (Limitations of the review)

The section appropriately acknowledges several limitations, including the lack of bias assessment and the UK-specific focus. However, the discussion could also briefly reflect on potential publication bias or language restrictions, given that only English-language studies were included.

Response: Please see response and revision in relation to Reviewer 1, Comment 1.

Reviewer 2, Comment 23

The identification of research gaps—such as the limited evidence on rural contexts and longer-term evaluations—is valuable. This section could be strengthened by more clearly highlighting specific priorities for future research, particularly regarding longitudinal studies and causal evaluations of interventions.

Response: We have added in the Conclusion (lines 598-599) a mention of this research gap that we identified.

Reviewer 2, Comment 24 (Conclusion)

The conclusion provides a clear summary of the key findings and emphasises the role of public attitudes in shaping the implementation of active travel interventions. The section is concise and appropriately aligned with the results of the review.

Response: Thank you for these positive comments.

Reviewer 2, Comment 25

The conclusion provides a concise summary of the study; however, it could be strengthened by more clearly reiterating the key insight of the review. In particular, emphasising the complexity of public attitudes toward active travel interventions and the importance of effective public engagement in policymaking would help reinforce the central contribution of the study and highlight its relevance for policy and practice.

Response: We have added a clause about the complexity of the issue (lines 594-595) which links to the following line about the critical role of public engagement in active travel and physical activity strategies.

Reviewer 2, Comment 26

The call for further research is appropriate; however, the conclusion may benefit from briefly highlighting practical implications for policymakers, particularly regarding communication strategies, stakeholder engagement, and long-term evaluation of interventions.

Response: The practical implications are mentioned (lines 595-600) and the point on longer-term evaluations has been added in response to Reviewer 2's Comment 23.

Reviewer 2, Comment 27

While the UK-specific focus of the review is acknowledged in the limitations section, the conclusion does not reflect on what this means for the applicability of the findings beyond the UK context. Given that active travel policy is an area of growing international interest, briefly acknowledging in the conclusion that findings may not be directly transferable to other national or policy contexts, due to differences in governance structures, built environments, and cultural attitudes, would provide a more balanced and appropriately cautious closing statement.

Response: The UK-specific scope of the review is now clearly articulated throughout the manuscript, including the title (lines 1-2), Strengths/Limitations summary content (lines 65-66), methods (Table 1), and Discussion/limitations (lines 506, 533-534 and 556). Therefore, we do not consider additional repetition in the Conclusion necessary.

Reviewer 2, Comment 28 (References)

The reference list is relevant, wide-ranging, and appropriately includes policy reports and government documents alongside academic literature, which is well-suited to the policy-oriented nature of this scoping review. However, some inconsistencies in formatting are noted across the list — for example, variation in the inclusion of issue numbers, page ranges, and DOIs, inconsistent presentation of journal titles (some abbreviated, others written in full), and occasional use of URLs where DOIs are available. A final check against BMJ Open's referencing requirements to ensure consistency across all citations would strengthen the presentation of an otherwise comprehensive and well-constructed reference list.

Response: Thanks for taking time to check the references. The citation of government and policy reports means that DOIs are not available for all references, and we have used a web link in these cases. inte

We have also carefully rechecked the reference list against BMJ Open's requirements and made minor changes. For example, reference styles were either corrected (for example in Winters, Buehler and Götschi line 662 we corrected the volume and issue number presentation) or harmonised styles between references where these were inconsistent previously (e.g. Cleland et al (line 847 and Panter et al line 811 journal title harmonised).

References

1. Porter A, Walker R, House D, Salway R, Dawson S, Ijaz S, de Vocht F, Jago R. Physical activity interventions in European primary schools: a scoping review to create a framework for the design of tailored interventions in European countries. Frontiers in Public Health. 2024;Volume 12 - 2024. doi: 10.3389/fpubh.2024.1321167
2. Porter A, Jago R, Robles LA, Cawley E, Rogers PJ, Ferriday D, Brunstrom JM. Investigating the psychology of eating after exercise — a scoping review. Journal of Nutritional Science. 2025;14:e12. doi: 10.1017/jns.2024.99
3. Garritty C, Gartlehner G, Nussbaumer-Streit B, King VJ, Hamel C, Kamel C, Affengruber L, Stevens A. Cochrane Rapid Reviews Methods Group offers evidence-informed guidance to conduct rapid reviews. Journal of Clinical Epidemiology. 2021;130:13-22.
4. Mak S, Thomas A. Steps for conducting a scoping review. Journal of Graduate Medical Education. 2022;14(5):565-7.

VERSION 2 - REVIEW

**Reviewer**	**1**
**Name**	**Andriyani, Fitria**
**Affiliation**	**Universitas Negeri Yogyakarta, Department of Sports Education**
**Date**	**09-May-2026**
**COI**	

The authors have adequately addressed all reviewer comments and revised the manuscript accordingly. The quality and clarity of the manuscript have improved substantially.

**Reviewer**	**2**
**Name**	**Zhandire, Tracy**
**Affiliation**	**University of KwaZulu-Natal College of Health Sciences, School of Nursing & Public Health**
**Date**	**12-May-2026**
**COI**	

The introduction is generally well written; however, some sentences are overly long and dense, particularly in the discussion of devolution and policy divergence. Consider simplifying or breaking up lengthy sentences to improve readability. For example, lines 102–109 contain multiple ideas within a single sentence structure, which may reduce clarity for readers unfamiliar with UK governance structures
The manuscript introduces "acceptability" appropriately; however, the discussion would benefit from a more critical conceptual framing. The current definition is relatively broad and could be strengthened by briefly acknowledging that public acceptability is dynamic, socially constructed, and potentially influenced by political, media, and socio-economic contexts.
The discussion of devolution is informative, but the explicit connection between devolved governance and public attitudes towards active travel interventions could be more clearly articulated. The manuscript explains policy divergence well, but it would strengthen the argument to explain more directly why devolution may shape public perceptions, support, or opposition differently across UK regions.
**Research gap justification**<br><br>The rationale for conducting the scoping review is generally convincing. However, the statement that "the full range of active travel interventions might be insufficiently captured and analysed in depth" would benefit from greater specificity regarding what dimensions are missing in existing reviews (e.g., geographic diversity, intervention types, socio-political responses, or methodological approaches). **Line 124 - 125**

**Scoping review justification**<br><br>The justification for using a scoping review methodology is appropriate and aligns with established scoping review guidance. Nonetheless, the authors may consider strengthening this section by explicitly stating why a scoping review was preferred over a systematic review, particularly in relation to the anticipated heterogeneity of evidence sources, study designs, and policy contexts.
The objectives are clearly presented and logically aligned with the research question. However, Objective 3 could be clarified grammatically for readability. The phrase "between nations and regions and socio-economic characteristics" is slightly awkward and may be revised for clarity.
The inclusion of consultation with previous collaborators during protocol development is valuable; however, the manuscript should clarify whether this consultation aligns with Arksey and O'Malley's optional consultation stage or whether it was conducted solely to refine the search strategy and objectives. This distinction would improve methodological transparency.
The manuscript refers readers to Table 1 and the protocol for eligibility criteria, but a concise summary of the key inclusion and exclusion criteria within the main text would improve readability and methodological completeness for readers who may not consult supplementary materials.
**Search strategy transparency**<br><br>The search strategy is generally well described and appropriately includes multiple databases and grey literature searching through Google Scholar. However, the authors may consider clarifying:<br><br>1. whether language restrictions were applied;<br>2. whether publication date limits were used; and<br>3. whether any geographical filters were implemented beyond the UK focus.<br><br>This would improve reproducibility and methodological transparency.
**Screening process clarification**<br><br>The screening process is described clearly, and the use of independent screening strengthens methodological credibility. However, the statement regarding "a sufficiently high degree of agreement" would benefit from clarification regarding whether any formal

inter-rater reliability measure (e.g., Cohen's kappa) was calculated. If not, the authors should state this explicitly.
The rationale for screening 30% of records independently is partially justified through reference to previous literature and rapid review guidance. However, because this is presented as a scoping review rather than a rapid review, the authors may consider strengthening the justification for why full dual screening was not undertaken.
The description of thematic analysis is appropriate; however, the authors may consider specifying whether the analysis was inductive, deductive, or hybrid in approach. This would strengthen analytical transparency.<br><br>**Reporting stage**<br><br>The reporting section is concise but somewhat brief. The authors may consider clarifying whether findings were synthesised narratively only or whether tabular/chart-based presentation methods were also used.
The patient and public involvement statement is clear and appropriate. However, given the policy-oriented nature of the review, the authors may consider briefly acknowledging whether stakeholder engagement beyond protocol consultation was considered or why it was not undertaken.
The exclusion criterion "Public outside the UK, even if intervention in UK" could be clarified for readability and conceptual precision. It is currently slightly ambiguous regarding cross-national or comparative studies involving UK populations.
The inclusion definition of active travel is appropriate and well referenced. However, the distinction between "everyday purposes" and "exercise or recreation" may require further clarification, particularly where studies involve mixed motivations for walking or cycling.<br><br>The authors may consider briefly clarifying how studies involving overlapping transport and recreational purposes were handled during screening.
The definition of "public attitudes" is comprehensive, but the wording is somewhat lengthy and difficult to follow. The phrase:<br><br>"Oriented by facts and/or emotions and prior beliefs" could potentially be simplified for readability.<br><br>Additionally, the exclusion criterion:<br><br>"Studies using attitudes solely/mainly to predict or explain active travel behaviour" would benefit from clarification regarding whether behavioural intention studies were excluded entirely or only where attitudes were not linked to intervention acceptability.

**Intervention definition**<br><br>The inclusion criterion for active travel interventions is appropriate; however, the exclusion example:<br><br>“policy only to reduce vehicular emissions” may be problematic because some environmental policies may indirectly promote active travel.<br><br>The authors may consider clarifying whether interventions with dual environmental and active travel objectives were included where active travel promotion formed part of the intervention rationale.
The devolution criterion is clear and appropriately justified. However, the table may benefit from explicitly clarifying whether studies using retrospective data spanning pre- and post-1999 periods were eligible if post-1999 findings were separately identifiable.
The exclusion of grey literature, conference proceedings, and unpublished studies should be more explicitly justified in the methods section given that scoping reviews often include broader evidence sources.<br><br>The authors may wish to explain:<br><br>1. why grey literature was excluded despite the policy-oriented nature of the review;<br>2. and how this exclusion may affect comprehensiveness or publication bias.
The opening paragraph appropriately summarises the number of included studies and supporting tables/figures. However, the statement:<br><br>“Descriptive statistics of key study features are in Table 2”, could be strengthened by briefly highlighting one or two key trends identified in the dataset. **Line 210 - 211**
The categorisation of interventions is clear and useful. Nonetheless, some intervention categories appear to overlap conceptually, particularly where interventions combine infrastructural and behavioural components. The manuscript may benefit from briefly clarifying how overlapping interventions were classified.
**Theme structure and analytical synthesis**<br><br>The thematic organisation is a major strength of the section. The three broad themes are coherent and well linked to the research objectives.

However, some subsections remain heavily descriptive. The manuscript would benefit from slightly stronger synthesis across studies rather than sequential reporting of findings from individual publications.<br><br>For example:<br><br>1. greater comparison between intervention types;<br>2. stronger discussion of tensions across social groups;<br>3. more explicit linking between attitudes, implementation stages, and policy implications.
The discussion of socio-economic and demographic differences is insightful. However, the finding regarding limited evidence on ethnicity is particularly important and deserves stronger critical reflection, especially given transport inequalities and representation concerns.<br><br>This could potentially be elevated into a more explicit evidence gap.
The “Interventions must be fair to all” theme is strong and policy relevant. However, the discussion could benefit from more explicit conceptual linkage to procedural fairness, distributive fairness, or equity frameworks within public health or transport policy literature.
The discussion of complexity is nuanced and compelling. The examples demonstrating divergent interpretations of the same intervention are particularly valuable.<br><br>However, the section may benefit from occasional synthesis sentences that consolidate the broader implications of these findings for policymakers and implementation science.
The media discussion is interesting and relevant but currently somewhat underdeveloped relative to its importance in shaping public attitudes.<br><br>The authors may consider:<br><br>1. expanding slightly on media framing effects;<br>2. or linking this more clearly to public trust, misinformation, or policy legitimacy.
1. Ensure consistent use of terminology such as:<br>o “Low traffic neighbourhoods” vs “low traffic areas”;<br>o “post-implementation” vs “post implementation.”<br>2. Some paragraphs are very long and could be broken into smaller units for readability.<br>3. Consider reducing repetition around congestion, driver resistance, and fairness concerns across multiple subsections.

The opening paragraph provides a strong synthesis of the review findings and effectively summarises the main themes emerging from the evidence base.

However, the paragraph is quite dense and lengthy. Consider breaking it into two shorter paragraphs to improve readability and reduce cognitive load for readers.

Evidence gaps and geographic exclusions

The manuscript appropriately identifies evidence gaps relating to Wales, North East England, rural contexts, and specific travel modes.

However, the discussion could more critically reflect on how these omissions may affect the transferability or representativeness of the review findings across devolved UK contexts.

The limitations section is transparent and appropriately acknowledges exclusion of non-English and grey literature due to resource constraints. However, given the policy-oriented nature of the review, the exclusion of grey literature may have omitted important local authority reports, transport consultations, and policy evaluations. The implications of this exclusion could be discussed more explicitly.

The statement:

"As a scoping review, the study does not assess potential bias..." is appropriate. However, the manuscript may benefit from clarifying whether omission of quality appraisal was a methodological choice consistent with the review aims or whether it reflects resource constraints.

The discussion demonstrates good integration with broader literature on cycling, public resistance, and transport policy. However, the discussion could be strengthened by engaging more explicitly with implementation science, behavioural policy, or public trust literature, particularly regarding policy acceptability and sustained public support.

The discussion of public cynicism and declining trust in governments is particularly relevant and insightful.

However, these concepts would benefit from deeper theoretical framing. The authors may consider engaging more directly with concepts such as:

1. procedural legitimacy;
2. institutional trust;
3. participatory governance; or
4. policy legitimacy.

The distinction between:<br><br>"decide-announce-defend" and "engage-deliberate-decide" is strong and policy relevant. However, the manuscript may benefit from briefly explaining these approaches for readers unfamiliar with participatory policymaking terminology.
The future research section appropriately identifies methodological and geographic gaps. However, the recommendation for more experimental or quasi-experimental studies could be further justified in relation to the specific challenges of measuring attitudinal change over time.
The conclusion succinctly summarises the key implications of the review and aligns well with the study objectives. However, the conclusion could be strengthened slightly by ending with a clearer statement regarding the broader implications for sustainable transport policy or public health strategy in the UK.

VERSION 2 - AUTHOR RESPONSE

General response from authors to reviewer:

We are grateful to the reviewer for their thorough reading of the manuscript and the considered comments. We have responded to each point below and indicated where we have revised the manuscript.

Please note all line references in author responses refer to the clean copy of the latest manuscript whereas reviewer's line references are to the previous version of the manuscript.

Comment 1: The introduction is generally well written; however, some sentences are overly long and dense, particularly in the discussion of devolution and policy divergence. Consider simplifying or breaking up lengthy sentences to improve readability. For example, lines 102–109 contain multiple ideas within a single sentence structure, which may reduce clarity for readers unfamiliar with UK governance structures.

Author response: Thank you for the comment about the introduction. We have made edits as suggested to the longer sentences (lines 75 and 95-96). We took this opportunity to break up and clarify the part on UK political devolution and policy divergence to aid readers unfamiliar with UK governance structures (lines 101-111).

Comment 2: The manuscript introduces "acceptability" appropriately; however, the discussion would benefit from a more critical conceptual framing. The current definition is relatively broad and could be strengthened by briefly acknowledging that public acceptability is dynamic, socially constructed, and potentially influenced by political, media, and socio-economic contexts.

Author response: We have addressed these points, citing relevant literature on complexity and on the acceptability of public health interventions (lines 91-95).

Comment 3: The discussion of devolution is informative, but the explicit connection between devolved governance and public attitudes towards active travel interventions could be more clearly articulated. The manuscript explains policy divergence well, but it would strengthen the argument to explain more directly why devolution may shape public perceptions, support, or opposition differently across UK regions.

Author response: We have made more explicit our point that devolution creates new, more localised contexts for public attitudes to interact with policymaking (lines 114-115) as well as policy divergence more generally. We have added a citation to illustrate this point with a paper that found differences between the UK's constituent nations in public acceptability towards Covid-19 control measures.

Comment 4 Research gap justification: The rationale for conducting the scoping review is generally convincing. However, the statement that "the full range of active travel interventions might be insufficiently captured and analysed in depth" would benefit from greater specificity regarding what dimensions are missing in existing reviews (e.g., geographic diversity, intervention types, socio-political responses, or methodological approaches). Line 124 – 125

Author response: Thanks for your compliment about the rationale. We have clarified the quoted sentence (lines 126-131) to indicate some of the dimensions we noted were missing when preparing the study protocol. These inform the research question and objectives too.

Comment 5 Scoping review justification: The justification for using a scoping review methodology is appropriate and aligns with established scoping review guidance. Nonetheless, the authors may consider strengthening this section by explicitly stating why a scoping review was preferred over a systematic review, particularly in relation to the anticipated heterogeneity of evidence sources, study designs, and policy contexts.

Author response: We have revised the text to make clearer the rationale for choosing a scoping review specifically (lines 138-142), citing Munn et al's article [1] that proposes indications for researchers deciding between a scoping or systematic review approach.

Comment 6: The objectives are clearly presented and logically aligned with the research question. However, Objective 3 could be clarified grammatically for readability. The phrase "between nations and regions and socio-economic characteristics" is slightly awkward and may be revised for clarity.

Author response: We have made a minor edit (lines 149-150) to make this line clearer while retaining the original research objectives we pre-registered in the protocol.

Comment 7: The inclusion of consultation with previous collaborators during protocol development is valuable; however, the manuscript should clarify whether this consultation aligns with Arksey and O'Malley's optional consultation stage or whether it was conducted solely to refine the search strategy and objectives. This distinction would improve methodological transparency.

Author response: We have revised this part to be more specific about the consultation purpose (lines 166-168). Reporting the purpose is recommended by Levac et al [2], who further developed Arksey & O'Malley's suggestion to consult.

Comment 8: The manuscript refers readers to Table 1 and the protocol for eligibility criteria, but a concise summary of the key inclusion and exclusion criteria within the main text would improve readability and methodological completeness for readers who may not consult supplementary materials.

Author response: We considered this recommendation carefully and understand that BMJ Open would place Table 1 in the main text and asks authors to do this as near as possible to where the table is first cited. This would make it easily available for readers to consult, so we consider it would be unnecessary duplication to add another summary into the main text.

Comment 9 Search strategy transparency: The search strategy is generally well described and appropriately includes multiple databases and grey literature searching through Google Scholar. However, the authors may consider clarifying:

1. whether language restrictions were applied;

2. whether publication date limits were used; and

3. whether any geographical filters were implemented beyond the UK focus.

This would improve reproducibility and methodological transparency.

Author comments: We consider the current wording supports reproducibility and transparency as we have provided a full copy of the main search strategy and limits applied, including the exact search syntax used. We think the language restrictions applied are already clear and prominent in the manuscript (in the Abstract, line 25; Table 1 – Source characteristics; and lines 546-548); and likewise the publication date limits (in the Abstract, lines 25-26; Table 1 – Concept 3; and line 222).

Comment 10 Screening process clarification: The screening process is described clearly, and the use of independent screening strengthens methodological credibility. However, the statement regarding "a sufficiently high degree of agreement" would benefit from clarification regarding whether any formal inter-rater reliability measure (e.g., Cohen's kappa) was calculated. If not, the authors should state this explicitly.

Author comments: We have now clarified that by "degree of agreement" we meant the proportionate agreement between the reviewers, which is the proportion of inclusion and exclusion decisions on which independent reviewers agreed, expressed as a proportion of overall decisions made (line 184-185). At title-abstract stage this proportion was ~96% and the disagreements were resolved with discussion between screeners to help calibrate the criteria. The vast majority of independent title-abstract screening decisions by the two reviewers were exclude-exclude. We understand that Cohen's kappa is not a suitable measure for analysing decisions with this level of skew, so we did not think it appropriate to conduct additional analyses of this type.

Comment 11: The rationale for screening 30% of records independently is partially justified through reference to previous literature and rapid review guidance. However, because this is presented as a scoping review rather than a rapid review, the authors may consider strengthening the justification for why full dual screening was not undertaken.

Author comments: Our approach sought to make the search broad and comprehensive enough for the research question but also feasible. We understand that this approach is a potential limitation of the review and we report this prominently in a number of places in the manuscript (Strengths/Limitations of study, lines 60-61; and 183-187). These parts mention our rationale that this decision was to balance considerations of feasibility with achieving an appropriate breadth and depth in the review. Our selection of 30% was benchmarked against published scoping review literature in public health [3, 4] and by taking the recommended rapid review independent screening rate of 20%

and exceeding it to 30% as we were performing a scoping review which had higher standards of rigour (lines 188-190).

Comment 12: The description of thematic analysis is appropriate; however, the authors may consider specifying whether the analysis was inductive, deductive, or hybrid in approach. This would strengthen analytical transparency.

Author comments: We have revised this section with greater detail of our hybrid method, which was to code inductively from the findings but only within the bounds of the research question and objectives (lines 203-204).

Comment 13 Reporting stage: The reporting section is concise but somewhat brief. The authors may consider clarifying whether findings were synthesised narratively only or whether tabular/chart-based presentation methods were also used.

Author comments: We have added further details of our analytical method (lines 201-206 and 208-209) to address this point.

Comment 14: The patient and public involvement statement is clear and appropriate. However, given the policy-oriented nature of the review, the authors may consider briefly acknowledging whether stakeholder engagement beyond protocol consultation was considered or why it was not undertaken.

Author comments: BMJ Open requires all submitted manuscripts to include a statement, even if only to state that no patient or public involvement was undertaken. While we do not mention our consultation with potential future users of the scoping review findings it is described earlier in the Methods section (lines 164-169).

Comment 15: The exclusion criterion "Public outside the UK, even if intervention in UK" could be clarified for readability and conceptual precision. It is currently slightly ambiguous regarding cross-national or comparative studies involving UK populations. The inclusion definition of active travel is appropriate and well referenced. However, the distinction between "everyday purposes" and "exercise or recreation" may require further clarification, particularly where studies involve mixed motivations for walking or cycling.

Author comments: In line with our study protocol and reviewer feedback in this comment we revised Table 1's definition of 'UK public' to clarify that comparative studies involving UK and non-UK populations were included. Our shared search strategy shows how this was operationalised for the search.

We think our exclusion definition on journey purposes is clearly reported as excluding journeys taken "solely for exercise or recreation". We have done this as our study has a public health focus, so we include only "everyday journeys" that could be shifted away from motorized transport modes to active travel modes for a physical activity and public health benefit. See Comment 16 response for how we handled this in practice.

Comment 16: The authors may consider briefly clarifying how studies involving overlapping transport and recreational purposes were handled during screening.

Author response: We've added a reference in Table 1 - Concept 2b to clarify that we looked at government policy intention and aims. We generally included studies if the intervention had a discernible active travel policy aim, even if recreation or public transport were also aims. For example, we included two studies about the Cambridgeshire busway infrastructural intervention [5, 6] as there was clear evidence of an active travel policy aim even though the intervention name does not initially suggest this.

Comment 17: The definition of "public attitudes" is comprehensive, but the wording is somewhat lengthy and difficult to follow. The phrase: "Oriented by facts and/or emotions and prior beliefs" could potentially be simplified for readability.

Author response: we have simplified Table 1 - Concept 2a for clarity while retaining the meaning we originally specified in our published protocol.

Comment 18: Additionally, the exclusion criterion: "Studies using attitudes solely/mainly to predict or explain active travel behaviour" would benefit from clarification regarding whether behavioural intention studies were excluded entirely or only where attitudes were not linked to intervention acceptability.

Author response: We think the criterion in Table 1 is reasonably clear that studies focusing "solely or mainly" on travel behaviour as an outcome or phenomenon of interest were excluded because our outcome/phenomenon was attitudes. By contrast we included studies that used travel behaviour to predict or explain attitudes to active travel if they satisfied the other inclusion criteria.

Comment 19 Intervention definition: The inclusion criterion for active travel interventions is appropriate; however, the exclusion example: "policy only to reduce vehicular emissions" may be problematic because some environmental policies may indirectly promote active travel. The authors may consider clarifying whether

interventions with dual environmental and active travel objectives were included where active travel promotion formed part of the intervention rationale.

Author response: Similarly to the responses to Comments 15 and 16, we included interventions with evidence of government policy intent to promote active travel and excluded interventions, such as London's Ultra Low Emission Zone, that are targeted at vehicular emissions. We excluded any intervention that had "no specific aim to promote physical activity" (see Table 1). We are aware that this has potential limitations that we mention in Discussion (lines 556-559), namely that public attitudes to active travel could be influenced by interventions or factors in ways not intended by governments.

Comment 20: The devolution criterion is clear and appropriately justified. However, the table may benefit from explicitly clarifying whether studies using retrospective data spanning pre- and post-1999 periods were eligible if post-1999 findings were separately identifiable.

Author response: We made a small edit (Table 1) to clarify that studies with "most or all" data pre-1999 were excluded.

Comment 21: The exclusion of grey literature, conference proceedings, and unpublished studies should be more explicitly justified in the methods section given that scoping reviews often include broader evidence sources. The authors may wish to explain:

1. why grey literature was excluded despite the policy-oriented nature of the review;

2. and how this exclusion may affect comprehensiveness or publication bias.

Author response: our rationale for excluding these sources is set out in the Strengths/Limitation box on the front page (lines 67-69) and Methods section (line 548); and in Discussion in the context of the potential effect of the exclusion (lines 546-552). See Comment 32 for further response to reviewer's point 2.

Comment 22: The opening paragraph appropriately summarises the number of included studies and supporting tables/figures. However, the statement: "Descriptive statistics of key study features are in Table 2", could be strengthened by briefly highlighting one or two key trends identified in the dataset. Line 210 – 211

Author response: We have added clarification (lines 215-217) that Table 2 summarises the content of the publication, intervention and public characteristics sections. We do

not believe that further summaries should be added to the text because Table 2 is itself a summary of the characteristics content (lines 222-285).

Comment 23: The categorisation of interventions is clear and useful. Nonetheless, some intervention categories appear to overlap conceptually, particularly where interventions combine infrastructural and behavioural components. The manuscript may benefit from briefly clarifying how overlapping interventions were classified.

Author response: We discuss the concept that interventions can combine multiple components: for example being both behavioural and infrastructural (lines 85-88; and 240-249); and the 'other' category for interventions that do not neatly fit into either category. For more common combinations of behavioural and infrastructural interventions we created separate categories, such as active travel to specific locations (lines 247-248). As well as explaining the categorisation we offer brief examples of interventions in each category (Figure 2).

Comment 24 Theme structure and analytical synthesis: The thematic organisation is a major strength of the section. The three broad themes are coherent and well linked to the research objectives. However, some subsections remain heavily descriptive. The manuscript would benefit from slightly stronger synthesis across studies rather than sequential reporting of findings from individual publications.

For example:

1. greater comparison between intervention types;

2. stronger discussion of tensions across social groups;

3. more explicit linking between attitudes, implementation stages, and policy implications.

Author response: Thanks for the comments about the thematic organisation. We do not agree that the manuscript does "sequential reporting of findings" because the results section is organised by theme. In the three examples highlighted we argue there is adequate synthesis and discussion of the points raised:

1. *Comparison of intervention types: our analysis points out that cycling is perceived as more exclusive and visible than other active travel modes (lines 326-332 and 465-469); and that walking is much less tangible (lines 473-475). We also highlight the importance of the infrastructural interventions as a perceived gap by the public (lines 460-463).*
2. *Tensions across social groups: we mention the evidence that drivers dislike active travel interventions and the specific reasons for this (lines 431-435); and the theme of tensions across social groups comes across clearly in the*

'Interventions must be fair to all' subtheme (lines 399-412) and the complexity subtheme (lines 496-504).

3. *Intervention stages and policy implications: we present the evidence about attitudes pre-intervention (lines 483-485); during interventions (lines 483-489); and post-intervention (lines 375-377; and 440-441). We draw this out as an evidence gap; and also discuss the policy implications of attitudes at different stages in the Discussion (lines 537-521; and 595-600).*

Comment 25: The discussion of socio-economic and demographic differences is insightful. However, the finding regarding limited evidence on ethnicity is particularly important and deserves stronger critical reflection, especially given transport inequalities and representation concerns. This could potentially be elevated into a more explicit evidence gap.

Author response: We have added specific mention to this gap in the Discussion section to make it more prominent and to be aligned with the other gaps identified (lines 608-609).

Comment 26: The "Interventions must be fair to all" theme is strong and policy relevant. However, the discussion could benefit from more explicit conceptual linkage to procedural fairness, distributive fairness, or equity frameworks within public health or transport policy literature.

Author response: we have revised the Discussion to highlight that the review findings include different aspects of fairness, such as the distinction between fair processes and fair outcomes. We have added a citation to an equity framework in transport policy (line 598).

Comment 27: The discussion of complexity is nuanced and compelling. The examples demonstrating divergent interpretations of the same intervention are particularly valuable. However, the section may benefit from occasional synthesis sentences that consolidate the broader implications of these findings for policymakers and implementation science.

Author response: Thanks for this compliment. We have added this to the Discussion section to make this part more coherent, referring back to the themes raised in the Introduction about intervention and complexity (line 575-577). See response to Comment 2 for this revision.

Comment 28: The media discussion is interesting and relevant but currently somewhat underdeveloped relative to its importance in shaping public attitudes.

The authors may consider:

1. expanding slightly on media framing effects;

2. or linking this more clearly to public trust, misinformation, or policy legitimacy.

Author response: We have revised this part (lines 532-533) in line with the second suggestion. We mention that the limitations of the media and consultation tools available to governments are a potential barrier to gaining common understanding between policymakers and all people affected by interventions.

Comment 29: Ensure consistent use of terminology such as:

- “Low traffic neighbourhoods” vs “low traffic areas”;
- “post-implementation” vs “post implementation.”

2. Some paragraphs are very long and could be broken into smaller units for readability.

3. Consider reducing repetition around congestion, driver resistance, and fairness concerns across multiple subsections.

Author response: in relation to 1 and 2 we have harmonised wording as suggested (lines 235, 394 and 427). We also revised the manuscript in one place to reduce ambiguity as this is discussing “areas with lower traffic” rather than designated “Low Traffic Neighbourhoods” (lines 370-371). Regarding point 3 we considered the instances of repetition of congestion, driver resistance and fairness concerns. They were very prevalent issues in the included literature and congestion, driver resistance and fairness arise in different ways and across themes so we believe this justifies their repetition across some of the themes.

Comment 30: The opening paragraph provides a strong synthesis of the review findings and effectively summarises the main themes emerging from the evidence base. However, the paragraph is quite dense and lengthy. Consider breaking it into two shorter paragraphs to improve readability and reduce cognitive load for readers.

Author response: in line with this suggestion, we have now divided this part into three paragraphs: the review purpose and publication characteristics (lines 518-523); thematic findings (lines 524-533) and evidence gaps (lines 534-541).

Comment 31 Evidence gaps and geographic exclusions: The manuscript appropriately identifies evidence gaps relating to Wales, North East England, rural contexts, and specific travel modes. However, the discussion could more critically reflect on how these omissions may affect the transferability or representativeness of the review findings across devolved UK contexts.

Author response: we have added critical reflection on these gaps (lines 534-537). Rural areas across the UK and Wales/North East England share separate commonalities (rural and post-industrial areas respectively), but these areas differ from the UK overall in important ways.

Comment 32: The limitations section is transparent and appropriately acknowledges exclusion of non-English and grey literature due to resource constraints. However, given the policy-oriented nature of the review, the exclusion of grey literature may have omitted important local authority reports, transport consultations, and policy evaluations. The implications of this exclusion could be discussed more explicitly.

Author response: We revised this part to discuss more explicitly the potential effects of omission, such as not including some relevant/useful evidence (lines 548-552). We have additionally cited public health methods literature about the advantages and disadvantages of including grey literature in this type of review. See Comments 9 and 21 cover related points to this comment.

Comment 33: The statement: "As a scoping review, the study does not assess potential bias..." is appropriate. However, the manuscript may benefit from clarifying whether omission of quality appraisal was a methodological choice consistent with the review aims or whether it reflects resource constraints.

Author response: This was a methodological choice and is similar to the issues raised in Comment 5. We presented our study rationale for a scoping review to answer the research question and address the identified literature gap (lines 137-142). Munn et al's guidance on indicating a scoping or systematic review [1] argues that scoping reviews do not usually assess potential bias unless evidence quality is a key part of the review objectives, which was not the case for our review.

Comment 34: The discussion demonstrates good integration with broader literature on cycling, public resistance, and transport policy. However, the discussion could be strengthened by engaging more explicitly with implementation science, behavioural policy, or public trust literature, particularly regarding policy acceptability and sustained public support.

Author response: Thanks for the compliment about the integration with broader literature. We think we have adequately linked the issues raised in the review with the types of literature the reviewer mentions while remaining focused on the review's scope. For example, we cite literature on growing post-intervention support (lines 599-600); and on complexity and the idea of interventions as 'events in systems' (line 602-604).

Comment 35: The discussion of public cynicism and declining trust in governments is particularly relevant and insightful. However, these concepts would benefit from deeper theoretical framing. The authors may consider engaging more directly with concepts such as:

1. procedural legitimacy;
2. institutional trust;
3. participatory governance; or
4. policy legitimacy.

Author response: Thanks for this suggestion. Our Discussion is currently focused on the key issues relevant to the research question and objectives. For example, the challenging context for active travel policymaking due to the longer term decline in trust in government (lines 582-589); the limitations of engagement tools (lines 572-575); and our reflection that this declining trust seems counterintuitive in the era of UK devolution, where policymaking is supposed to be more responsive to local needs and more accessible to people affected (lines 578-582). The concepts the reviewer raises are very interesting but we consider beyond the scope of our paper.

Comment 36: The distinction between: "decide-announce-defend" and "engage-deliberate-decide" is strong and policy relevant. However, the manuscript may benefit from briefly explaining these approaches for readers unfamiliar with participatory policymaking terminology.

Author response: We have revised this part as recommended (lines 589-594).

Comment 37: The future research section appropriately identifies methodological and geographic gaps. However, the recommendation for more experimental or quasi-experimental studies could be further justified in relation to the specific challenges of measuring attitudinal change over time.

Author response: We have revised this part as suggested (lines 613-615) and cited a recent paper on this issue.

Comment 38: The conclusion succinctly summarises the key implications of the review and aligns well with the study objectives. However, the conclusion could be strengthened slightly by ending with a clearer statement regarding the broader implications for sustainable transport policy or public health strategy in the UK.

Author response: We have added draft closing lines as suggested (lines 629-632).

References

1. Munn Z PM, Stern C, Tufanaru C, McArthur A, Aromataris E. Systematic review or scoping review? Guidance for authors when choosing between a systematic or scoping review approach. BMC Medical Research Methodology. 2018;18(1). doi: 10.1186/s12874-018-0611-x

2. Levac D, Colquhoun, Heather, O'Brien, Kelly K. Scoping studies: advancing the methodology. Implementation Science. 2010;5(1):69. doi: 10.1186/1748-5908-5-69

3. Porter A, Walker R, House D, Salway R, Dawson S, Ijaz S, de Vocht F, Jago R. Physical activity interventions in European primary schools: a scoping review to create a framework for the design of tailored interventions in European countries. Frontiers in Public Health. 2024;Volume 12 - 2024. doi: 10.3389/fpubh.2024.1321167

4. Porter A, Jago R, Robles LA, Cawley E, Rogers PJ, Ferriday D, Brunstrom JM. Investigating the psychology of eating after exercise — a scoping review. Journal of Nutritional Science. 2025;14:e12. doi: 10.1017/jns.2024.99

5. Kesten JM, Guell, Cornelia, Cohn, Simon, Ogilvie, David. From the concrete to the intangible: understanding the diverse experiences and impacts of new transport infrastructure. International Journal of Behavioral Nutrition and Physical Activity. 2015;12(1). doi: 10.1186/s12966-015-0230-4

6. Kesten JM, Cohn S, Ogilvie D. The contribution of media analysis to the evaluation of environmental interventions: the commuting and health in Cambridge study. BMC public health. 2014;14:482. doi: 10.1186/1471-2458-14-482
