# Supplementary material for: Scoping review of public attitudes to government active travel interventions in the UK
Source: BMJ Open. 2026 Aug 14;16(8):e115598. doi: 10.1136/bmjopen-2025-115598 (PMC13479531; doi:10.1136/bmjopen-2025-115598)
Supplement: Supplementary file 2 [file bmjopen-16-8-s001.docx]

**Appendix 1: Sample search strategy for Medline database (6 Feb. 2025)**

| **Concept 1: active travel** | | |
| --- | --- | --- |
| 1 | active* travel*.tw,kf. | 682 |
| 2 | active transport.tw,kf. | 8485 |
| 3 | active commut*.tw,kf. | 674 |
| 4 | (active travel adj3 school).tw,kf. | 107 |
| 5 | walk*.tw,kf. | 163080 |
| 6 | bicycl*.tw,kf. | 29524 |
| 7 | bike*.tw,kf. | 3469 |
| 8 | cyclist*.tw,kf. | 6789 |
| 9 | cycling.tw,kf. | 91330 |
| 10 | cycle*.tw,kf. | 788491 |
| 11 | wheel*.tw,kf. | 27801 |
| 12 | (non-motorized or non-motorised).tw,kf. | 359 |
| 13 | exp Bicycling/ | 13563 |
| 14 | exp Pedestrians/ | 1728 |
| 15 | or 1:14 | 295258 |
|  | | |
| **Concept 2: active travel interventions** | | |
| 16 | Intervention*.tw,kf. | 1546727 |
| 17 | (policy or policies).tw,kf. | 407098 |
| 18 | program*.tw,kf. | 1205561 |
| 19 | scheme*.tw,kf. | 175939 |
| 20 | project*.tw,kf. | 448658 |
| 21 | (street* design* or (street adj3 design)).tw,kf. | 142 |
| 22 | (road* design* or (road adj3 design)).tw,kf. | 454 |
| 23 | (street* adj3 space).tw,kf. | 83 |
| 24 | (road* adj3 space).tw,kf. | 126 |
| 25 | (20mph or 20 mph or twenty mile* per hour).tw,kf. | 116 |
| 26 | (cycl* adj3 proficien*).tw,kf. | 38 |
| 27 | (traffic adj3 enforc*).tw,kf. | 280 |
| 28 | ((campaign* adj3 walk*) or (campaign* adj3 cycle*) or (campaign* adj3 wheel*)).tw,kf. | 55 |
| 29 | ((increas* adj3 walk*) or (increas* adj3 cycle*) or (increas* adj3 wheel*)).tw,kf. | 17298 |
| 30 | ((promot* adj3 walk*) or (promot* adj3 cycle*) or (promot* adj3 wheel*)).tw,kf. | 6406 |
| 31 | ((encourag* adj3 walk*) or (encourag* adj3 cycle*) or (encourag* adj3 wheel*)).tw,kf. | 470 |
| 32 | ((impact* adj3 walk*) or (impact* adj3 cycle*) or (impact* adj3 wheel*)).tw,kf. | 3326 |
| 33 | ((start* adj3 walk*) or (start* adj3 cycle*) or (start* adj3 wheel*)).tw,kf. | 2974 |
| 34 | ((habit* adj3 walk*) or (habit* adj3 cycle*) or (habit* adj3 wheel*)).tw,kf. | 691 |
| 35 | ((shift* adj3 walk*) or (shift* adj3 cycle*) or (shift* adj3 wheel*)).tw,kf. | 1052 |
| 36 | ((rent* adj3 bicycl*) or (shar* adj3 bicycl*) or (dock* adj3 bicycl*)).tw,kf. | 187 |
| 37 | ((rent* adj3 cycl*) or (shar* adj3 cycl*) or (dock* adj3 cycl*)).tw,kf. | 967 |
| 38 | pedestrianis*.tw,kf. | 13 |
| 39 | e-assist*.tw,kf. | 149 |
| 40 | (e-bike* or e-bicycle*).tw,kf. | 218 |
| 41 | ((loan* adj3 bicycle*) or (loan* adj3 cycle*)).tw,kf. | 5 |
| 42 | walk* school bus*.tw,kf. | 28 |
| 43 | (safe* adj3 school*).tw,kf. | 1506 |
| 44 | low traffic.tw,kf. | 280 |
| 45 | "active travel zone*".tw,kf. | 0 |
| 46 | "active travel area*".tw,kf. | 0 |
| 47 | "active travel neighborhood*".tw,kf. | 1 |
| 48 | Health Promotion/ | 84625 |
| 49 | Health Knowledge, Attitudes, Practice/ | 134587 |
| 50 | or 16:49 | 3473135 |
|  | | |
| **Concept 3: public attitudes** | | |
| 51 | attitude*.tw,kf. | 215801 |
| 52 | opinion*.tw,kf. | 143851 |
| 53 | support*.tw,kf. | 2186928 |
| 54 | perception*.tw,kf. | 380624 |
| 55 | sentiment*.tw,kf. | 6750 |
| 56 | oppos*.tw,kf. | 271438 |
| 57 | accepta*.mp. | 399946 |
| 58 | belief*.tw,kf. | 116576 |
| 59 | Public Opinion/ | 20389 |
| 60 | Attitude/ | 55314 |
| 61 | or 51:60 | 3383384 |
|  | | |
| **Limits: UK only [1]; and publication timeframe** | | |
| 62 | exp United Kingdom/ | 402346 |
| 63 | (UK or United Kingdom or Britain or Wales or Scotland or Northern Ireland or England or English or Scottish or Welsh or Irish).mp. [mp=title, book title, abstract, original title, name of substance word, subject heading word, floating sub-heading word, keyword heading word, organism supplementary concept word, protocol supplementary concept word, rare disease supplementary concept word, unique identifier, synonyms, population supplementary concept word, anatomy supplementary concept word] | 2302900 |
| 64 | (english not ((published or publication* or translat* or written or language* or speak* or literature or citation*) adj5 english)).ti,ab. | 139480 |
| 65 | (gb or "g.b." or britain* or (british* not "british columbia") or uk or "u.k." or united kingdom* or (england* not "new england") or northern ireland* or northern irish* or scotland* or scottish* or ((wales or "south wales") not "new south wales") or welsh*).ti,ab,jw,in. | 2638817 |
| 66 | (bath or "bath's" or ((birmingham not alabama*) or ("birmingham's" not alabama*) or bradford or "bradford's" or brighton or "brighton's" or bristol or "bristol's" or carlisle* or "carlisle's" or (cambridge not (massachusetts* or boston* or harvard*)) or ("cambridge's" not (massachusetts* or boston* or harvard*)) or (canterbury not zealand*) or ("canterbury's" not zealand*) or chelmsford or "chelmsford's" or chester or "chester's" or chichester or "chichester's" or coventry or "coventry's" or derby or "derby's" or (durham not (carolina* or nc)) or ("durham's" not (carolina* or nc)) or ely or "ely's" or exeter or "exeter's" or gloucester or "gloucester's" or hereford or "hereford's" or hull or "hull's" or lancaster or "lancaster's" or leeds* or leicester or "leicester's" or (lincoln not nebraska*) or ("lincoln's" not nebraska*) or (liverpool not (new south wales* or nsw)) or ("liverpool's" not (new south wales* or nsw)) or ((london not (ontario* or ont or toronto*)) or ("london's" not (ontario* or ont or toronto*)) or manchester or "manchester's" or (newcastle not (new south wales* or nsw)) or ("newcastle's" not (new south wales* or nsw)) or norwich or "norwich's" or nottingham or "nottingham's" or oxford or "oxford's" or peterborough or "peterborough's" or plymouth or "plymouth's" or portsmouth or "portsmouth's" or preston or "preston's" or ripon or "ripon's" or salford or "salford's" or salisbury or "salisbury's" or sheffield or "sheffield's" or southampton or "southampton's" or st albans or stoke or "stoke's" or sunderland or "sunderland's" or truro or "truro's" or wakefield or "wakefield's" or wells or westminster or "westminster's" or winchester or "winchester's" or wolverhampton or "wolverhampton's" or (worcester not (massachusetts* or boston* or harvard*)) or ("worcester's" not (massachusetts* or boston* or harvard*)) or (york not ("new york*" or ny or ontario* or ont or toronto*)) or ("york's" not ("new york*" or ny or ontario* or ont or toronto*))))).ti,ab,in. | 1915768 |
| 67 | (aberdeen or "aberdeen's" or dundee or "dundee's" or edinburgh or "edinburgh's" or glasgow or "glasgow's" or inverness or (perth not australia*) or ("perth's" not australia*) or stirling or "stirling's").ti,ab,in. | 281812 |
| 68 | (armagh or "armagh's" or belfast or "belfast's" or lisburn or "lisburn's" or londonderry or "londonderry's" or derry or "derry's" or newry or "newry's").ti,ab,in. | 37743 |
| 69 | (bangor or "bangor's" or cardiff or "cardiff's" or newport or "newport's" or st asaph or "st asaph's" or st davids or swansea or "swansea's").ti,ab,in. | 77808 |
| 70 | or 62:69 | 4983327 |
| 71 | (exp africa/ or exp americas/ or exp antarctic regions/ or exp arctic regions/ or exp asia/ or exp australia/ or exp oceania/) not (exp United Kingdom/ or europe/) | 3528714 |
| 72 | 70 not 71 | 4646876 |
| 73 | 15 and 50 and 61 and 72 | 2024 |
| 74 | limit 73 to yr="1999-Current" | **1916** |

**References**

1.	Lynda Ayiku PL, Tom Hudson, Jenny Craven, Elizabeth Barrett, Amy Finnegan, Rachel Adams. The medline UK filter: development and validation of a geographic search filter to retrieve research about the UK from OVID medline. Health Information & Libraries Journal. 2017;34(3). doi: 10.1111/hir.12187
