# Supplementary material for: Scoping review of public attitudes to government active travel interventions in the UK
Source: BMJ Open. 2026 Aug 14;16(8):e115598. doi: 10.1136/bmjopen-2025-115598 (PMC13479531; doi:10.1136/bmjopen-2025-115598)
Supplement: Supplementary file 3 [file bmjopen-16-8-s002.docx]

**Table 2: Key features of included publications, grouped by how public attitudes analysed**

| **People affected**  **(number of publications)** | **Paper ID** | **Study design** | **Geography** | **Intervention name (detail) or description if no name**   - Type/s of intervention | **Active Travel mode/s** | **Public and attitudes of interest** | **Data type (sample size/s)** | **Reported dates of intervention and data collection** |
| --- | --- | --- | --- | --- | --- | --- | --- | --- |
| **People using streets or areas affected**  **(n=10)** | Barr 2021 [58] | Qualitative | South West England | Shared street spaces   - Road design - Social and behavioural - Communications | Walking; Cycling | Views of people who travel in intervention area via any mode | Workshop attendee discussion  (participant n=96) | *Intervention*: not reported  *Data*: January-June 2017 |
|  | Caris 2024 [59] | Qualitative | London | Low Traffic Neighbourhoods, Mini Holland interventions, Streetspace and Liveable Neighbourhoods   - Road design - Within area travel (e.g. Low Traffic Neighbourhoods) | Walking; Cycling | People who have experienced the interventions in a personal or professional capacity | Interviews  (n=15; 6 of whom had personal experience) | *Interventions*: Spring 2020 onwards  *Data*: May-August 2022 |
|  | Cleland 2021 [60] | Qualitative | Northern Ireland | 20mph speed limit pilot   - Legislation, policy and/or regulation - Social and behavioural - Communications - Enforcement | Walking; Cycling | People exposed to the intervention due to living, working, socialising and/or travelling through or to the area | Focus groups  (participant n=60; 9 focus groups) | *Intervention :* February 2016  Data: July-December 2018 |
|  | Harrington 2024 [61] | Qualitative | Scotland | Shawlands Bike Bus   - Social and behavioural - Communications | Cycling; Wheeling | Attitudes of road and street users along the route affected by the intervention | Survey responses  (n=183) | *Intervention*: October 2021 onwards  *Data*: June 2023 |
|  | Jepson 2022 [62] | Mixed | Northern Ireland; Scotland | 20mph speed limits   - Legislation, policy and/or regulation - Social and behavioural - Communications - Enforcement | Walking; Cycling | People potentially exposed to the interventions in two cities, including public and professionals (e.g. bus and taxi drivers) | Focus groups  (Edinburgh participant n=99; 15 groups)  (Belfast participant n=60; 9 groups) | *See Cleland 2021 and Williams 2022 publications* |
|  | Lunetto 2023 [63] | Qualitative | London | Hackney and Lambeth Streetspace interventions   - Road design - Legislation, policy and/or regulation - Enforcement - Location specific travel - Within area travel (e.g. Low Traffic Neighbourhoods) | Walking; Cycling | Stakeholders, including residents and public, exposed to or affected by the interventions; and people contributing to an online consultation platform about the interventions | Interviews  (n=21; including 10 community groups/residents)  Online comments  (n=885) | *Intervention*: from May 2020  *Data*: May-August 2021 (interviews); February 2021 (online comments) |
|  | Melia 2018 [64] | Mixed | South East exc. London | Brighton Old Town traffic improvement scheme   - Road design - Within area travel (e.g. Low Traffic Neighbourhoods) | Walking | People exposed to intervention, both residents and those using the street | Survey responses (2013 n=280; 2015 weekday participants n=219;  2015 weekend participants n=218) | *Intervention*: consultation 2012, changes made August 2014 and May 2015  *Data*: June 2013 and June 2015 |
|  | Pritchett 2024 [65] | Qualitative | West Midlands | Kings Heath and Moseley LTNs   - Road design - Legislation, policy and/or regulation - Within area travel (e.g. Low Traffic Neighbourhoods) | Walking; Cycling | People exposed to the intervention | Online consultation platform responses  (Pre-intervention n=3751; post-intervention n=791) | *Intervention:*  September-November 2020  *Data*: consultations July-November 2020 and February-April 2021 |
|  | Semple 2023 [66] | Quantitative | Scotland | Edinburgh City Centre Transformation   - Road design - Within area travel (e.g. Low Traffic Neighbourhoods) | Walking; Cycling | People who use the city centre around the intervention area | Survey responses  (n=303) | *Intervention*: ~2019  *Data*: January-February 2020 |
|  | Williams 2022  [67] | Quantitative | Scotland | Edinburgh city 20mph speed limit   - Legislation, policy and/or regulation - Social and behavioural - Communications - Enforcement | Walking; Cycling | People in intervention areas | Survey responses  (n=2253) | *Intervention*: phased introduction by area July 2016-March 2018  *Data*: started August 2017 (for two areas) and March 2018 (one area) with collection at baseline, 6 months, and 12 months |
| **Residents**  **(n=4)** | Aldred 2019a [68] | Mixed | London | Mini-Holland programme   - Road design - Within area travel (e.g. Low Traffic Neighbourhoods) | Walking; Cycling | Residents from intervention areas | Survey  (n=2128; *54% control areas, 46% intervention areas)* | *Intervention*: 2016-17 onwards  *Data*: baseline May-June 2016; Wave 1 May-June 2017 |
|  | Aldred 2019b [69] | Quantitative | London | Mini-Holland programme   - Road design - Within area travel (e.g. Low Traffic Neighbourhoods) | Walking; Cycling | Residents before and during intervention | Survey  (Baseline n=3435;  Wave 1 n=1712) | *Intervention*: 2016-17 onwards  *Data*: baseline May-June 2016; Wave 1 May-June 2017 |
|  | Macniven 2024 [70] | Qualitative | Scotland | Dennistoun Spaces for People (Low Traffic Neighbourhood)   - Road design - Within area travel (e.g. Low Traffic Neighbourhoods) | Walking | Residents, either exposed to the intervention or those in higher traffic comparison areas | Focus groups  (n=20)  Follow-up interviews (n=6) | *Intervention:* not reported but was during Covid-19 pandemic  *Data*: not reported |
|  | Whelan 2024 [71] | Mixed | West Midlands | Kings Heath Low Traffic Neighbourhood   - Road design - Legislation, policy and/or regulation - Within area travel (e.g. Low Traffic Neighbourhoods) | Walking; Cycling | Residents exposed to intervention | Survey respondents (n=210) | *Intervention:* phase 1 2020; further phase due 2024  *Data*: November-December 2023 |
| **Commuters**  **(n=3)** | Audrey 2015 [72] | Qualitative | South West England | Walk to Work   - Social and behavioural - Communications - Location specific travel | Walking | Employee attitudes to a potential walking intervention via employers’ views | Interviews  (n=30, across 26 workplaces) | *Intervention*: trial during 2012  *Data*: not specifically reported, but during trial |
|  | Kesten 2014 [73] | Mixed | East of England | Cambridgeshire Guided Busway   - Road design | Walking; Cycling | People who travel to work in the intervention area; and newspaper and social media content about the intervention | Interviews  (n=38)  Newspaper articles  (n=363) | *Intervention*: ready for use August 2011  *Data*: February-June 2013 (interviews); media content covers October 2004 to November 2012 |
|  | Kesten 2015 [74] | Qualitative | East of England | Cambridgeshire Guided Busway   - Road design | Walking; Cycling | People who travel to work in the intervention area | Interviews  (n=38) | *Intervention*: ready for use August 2011  *Data*: February-June 2013 |
| **Parents**  **(n=2)** | Brown 2024 [75] | Quantitative | England | Promotion of active travel to school   - Road design - Social and behavioural - Communications - Location specific travel - Within area travel (e.g. LTNs) | Walking; Cycling; Wheeling | Parents in potential intervention areas about vehicular emissions reduction measures around schools | Survey respondents  (n=1441) | *Intervention*: prospective or hypothetical  *Data*: April-July 2021 |
|  | Nikitas 2019 [76] | Qualitative | Yorkshire & Humber | Shipley and Wycliffe Walking School Buses (primary schools with ~5-11 year olds)   - Social and behavioural - Communications | Walking | Parents in schools considering the feasibility and acceptability of the intervention | Focus groups  (participant n=25; 4 groups) | *Intervention*: prospective or hypothetical  *Data*: not reported |
| **Transport stakeholders**  **(n=3)** | Aldred 2019c [77] | Mixed | England | Other: Public funding and investment in cycling | Cycling | Public attitudes covered with transport intervention stakeholders (e.g. transport officers, consultants, academics, advocates) | Survey responses (n=413)  Interviews (n=7) | *Intervention*: prospective or hypothetical  *Data*: April-May 2016 |
|  | Lawlor 2023 [10] | Qualitative | UK studies included | Environmental interventions, including in UK   - Road design | Walking; Cycling | Review of evidence about stakeholders’ experiences of the intervention (e.g. planners, officers, public health professionals, advocacy groups, and policymakers) including public attitude considerations | Included studies  (n=35) | *Intervention*: not applicable  *Data*: systematic review searches April and November 2020 |
|  | Xenias 2013 [78] | Mixed | All-UK | Other: General descriptions of prospective active travel and modal shift interventions | Walking; Cycling | UK residents; and transport experts’ views on public attitudes (industry, government, interest groups, and academic) | Survey responses  (Residents n=40; expert n=53) | *Intervention*: prospective or hypothetical  *Data*: summer to autumn 2010 |
| **Samples of public**  **(n=2)** | Toumpakari 2023 [32] | Qualitative | England | Prospective active travel interventions covering economic, town planning, choice guiding, and information/education provision   - Road design - Legislation, policy and/or regulation - Social and behavioural - Communications - Facilitating access to bicycles | Cycling | Sample of public; and local and national public health policymakers | Interviews  (n=40; 50% public) | *Intervention*: prospective or hypothetical  *Data*: May 2021 (public members); October 2021-January 2022 (policymakers) |
|  | Tapp 2016 [79] | Quantitative | All-GB; South West England | Other: General descriptions of interventions to increase cycling | Cycling | GB residents | Survey responses (2010 n=3855; 2013 n=4022; 2013 additional fieldwork in Bristol n=507) | *Intervention*: prospective or hypothetical  *Data*: April-May 2010 and May-June 2013 |
| **Active travellers (pedestrians and cyclists)**  **(n=1)** | Jones 2012 [80] | Mixed | North West England; Yorkshire & Humber; East Midlands | Prospective interventions to promote active travel   - Road design | Walking; Cycling | Subsample of participants from a walking and cycling study invited to further research about active travel interventions | Survey statement rankings  (Participant n=25) | *Intervention*: prospective or hypothetical  *Data*: March-April 2011 |
| **Drivers**  **(n=1)** | Tapp 2015 [81] | Quantitative | All-GB | 20mph limits (signs only)   - Social and behavioural - Communications | Walking; Cycling | Randomly drawn respondents from an online panel, from whom a sample of self-reported drivers of motor vehicles sampled | Survey respondents  (n=2297; effective sample size 2219) | *Intervention*: prospective or hypothetical  Data: July 2013 |
